# Perceptual Disorders After Stroke: A Scoping Review of Interventions

**DOI:** 10.1161/STROKEAHA.121.035671

**Published:** 2022-03-18

**Authors:** Christine Hazelton, Kris McGill, Pauline Campbell, Alex Todhunter-Brown, Katie Thomson, Donald J. Nicolson, Joshua D. Cheyne, Charlie Chung, Liam Dorris, David C. Gillespie, Susan M. Hunter, Marian C. Brady

**Affiliations:** 1 Nursing, Midwifery and Allied Health Professions Research Unit, Glasgow Caledonian University, United Kingdom (C.H., K.M., P.C., A.T.-B., K.T., M.C.B.).; 2 Stroke Survivor Representative, United Kingdom (D.J.N.).; 3 Cochrane Stroke Group, University of Edinburgh, United Kingdom (J.D.C.).; 4 Queen Margaret Hospital, National Health Service (NHS) Fife, United Kingdom (C.C.).; 5 Paediatric Neurosciences, Royal Hospital for Children, NHS Greater Glasgow and Clyde, United Kingdom (L.D.).; 6 Department of Clinical Neurosciences, Royal Infirmary of Edinburgh, NHS Lothian, United Kingdom (D.C.G.).; 7 School of Allied Health Professions, Keele University, United Kingdom (S.M.H.).

**Keywords:** auditory perception, perceptual disorder, review, somatosensory disorders, stroke, touch perception, visual perception

## Abstract

Perceptual disorders relating to hearing, smell, somatosensation, taste, touch, and vision commonly impair stroke survivors’ ability to interpret sensory information, impacting on their ability to interact with the world. We aimed to identify and summarize the existing evidence for perceptual disorder interventions poststroke and identify evidence gaps. We searched 13 electronic databases including MEDLINE and Embase and Grey literature and performed citation tracking. Two authors independently applied a priori–defined selection criteria; studies involving stroke survivors with perceptual impairments and interventions addressing those impairments were included. We extracted data on study design, population, perceptual disorders, interventions, and outcomes. Data were tabulated and synthesized narratively. Stroke survivors, carers, and clinicians were involved in agreeing definitions and organizing and interpreting data. From 91 869 records, 80 studies were identified (888 adults and 5 children); participant numbers were small (median, 3.5; range, 1–80), with a broad range of stroke types and time points. Primarily focused on vision (34/80, 42.5%) and somatosensation (28/80; 35.0%), included studies were often case reports (36/80; 45.0%) or randomized controlled trials (22/80; 27.5%). Rehabilitation approaches (78/93; 83.9%), primarily aimed to restore function, and were delivered by clinicians (30/78; 38.5%) or technology (28/78; 35.9%; including robotic interventions for somatosensory disorders). Pharmacological (6/93; 6.5%) and noninvasive brain stimulation (7/93; 7.5%) approaches were also evident. Intervention delivery was poorly reported, but most were delivered in hospital settings (56/93; 60.2%). Study outcomes failed to assess the transfer of training to daily life. Interventions for stroke-related perceptual disorders are underresearched, particularly for pediatric populations. Evidence gaps include interventions for disorders of hearing, taste, touch, and smell perception. Future studies must involve key stakeholders and report this fully. Optimization of intervention design, evaluation, and reporting is required, to support the development of effective, acceptable, and implementable interventions.

**Registration:**

URL: https://www.crd.york.ac.uk/PROSPERO/; Unique identifier: CRD42019160270.

Perception is our ability to understand and organize information from our sensory systems: hearing, smell, somatosensation, taste, touch, and vision. Perceptual disorders are frequently undetected^1,2^ but may affect up to 74% of stroke survivors^3–5^ and persist for months or years post-onset.^3,6,7^ Perceptual disorders impact on stroke survivors’ ability to make sense of and interact with their environment, through recognition, differentiation, organization, and integration of sensory information,^8,9^ impeding recovery and rehabilitation,^10^ self-care,^11^ and independence in everyday activities.^3,12^

Assessment and management of perceptual problems poststroke is complex, due to the range of sensory systems and specialisms involved. Significant variability exists in care provision,^13^ pathways,^1,13^ and stroke team training on perception and terminology.^2,14^ While stroke guidelines refer to perception, treatment recommendations focus on specific domains, and guidance on intervention selection or delivery is limited.^15–17^ Intervention research is a priority for stroke survivors, carers, and health care professionals.^18,19^

Previous intervention evidence reviews are fragmented, addressing single sensory domains,^20^ specific anatomic areas,^21^ individual perceptual disorders,^22^ or specific interventions,^23^ while others include mixed populations inclusive of nonperceptual disorders or nonstroke etiologies.^24,25^ An accessible, comprehensive, up-to-date evidence review relevant to stroke survivors, carers, and clinicians is required. We aimed to identify, map, and synthesize evidence relating to perceptual disorder interventions poststroke in a scoping review, providing a broad overview of the evidence and identifying research gaps.

## Methods

There is much variation in the meaning assigned to the term perception: it varies in relation to definition, delineation from sensation and cognition, and included disorders; it also varies across senses, clinical specialisms, and time. We defined perception as “specific mental functions of recognizing and interpreting sensory stimuli”^26^ and applied it across disorders relating to hearing, smell, somatosensation (including proprioception), taste, touch, and vision (including visuospatial; see Methods S1 for definitions).

Our scoping review followed a predefined protocol (CRD42019160270), established methodology,^27,28^ and relevant reporting guidelines.^29^ Scoping review methodology provides a structured, rigorous approach to providing an overview of a range of evidence, research gaps, and future research priorities.^27,28^ Thirteen databases (including MEDLINE, Embase, and CINAHL), specialized resources, and trial registers were searched (inception to February 7, 2020). To address the breadth of included sensory domains and complex perceptual terminology, our multidisciplinary research team worked with stakeholders (stroke survivors and carers, n=5; experienced clinicians with expertise in perceptual disorders, n=4) and an information specialist to develop a peer-reviewed search^30^ (Methods S1). Extensive supplementary searching included backward and forward citation tracking (last search: November 24, 2020; Methods S1). No language or date limitations were applied. We included studies where participants had poststroke perceptual disorders and explored interventions that targeted that disorder. We included all age groups, stroke types, and settings.

Two reviewers independently screened abstracts and full texts. We anticipated challenges in the application of our perceptual disorder inclusion criteria: where uncertainties arose, a third (clinical expert) reviewer was consulted. Data were charted and categorized by 1 reviewer using predefined, piloted forms and checked by a second, with input from clinical experts as required. Extracted data included study design, participant demographics, intervention details (using the Template for Intervention Description and Replication [TIDieR] checklist),^31^ and outcome measurements. Where studies recruited mixed participant populations, stroke- and perception-specific data were extracted, where possible. Extensive data categorization profiled the complex disorders and interventions’ distinguishing features (Table S1). Intervention categorization used an established taxonomy,^32,33^ including pharmacological, noninvasive brain stimulation (NIBS; such as transcranial direct current stimulation), or rehabilitation. Rehabilitation interventions were subcategorized as restitution (direct training of the impaired function), compensation (via training of or using a spared function), substitution (use of an external device or modification),^34^ or a combination of these approaches. All categorizations were checked by a third reviewer and considered the body functions (impairments) the intervention targeted, as stated by the primary research teams; we made no assumptions about biological mechanisms at play.

We categorized outcomes used by the primary researchers to measure intervention effectiveness. We extracted verbatim summaries of individual study findings. As the aim was to provide an overview of the scope of research, rather than judge the quality of evidence for a specific intervention, no assessment of methodological quality or detailed aggregation of findings was conducted.^27^

Data were collated and tabulated. A narrative account was organized by sense and intervention approach. Our stakeholder group contributed to this process and data interpretation^35,36^ (Methods S2). The appropriate guidance was used to report the review (Methods S3). The review data are available from the corresponding author upon reasonable request.

## Results

### Results of the Search

**Figure 1.**
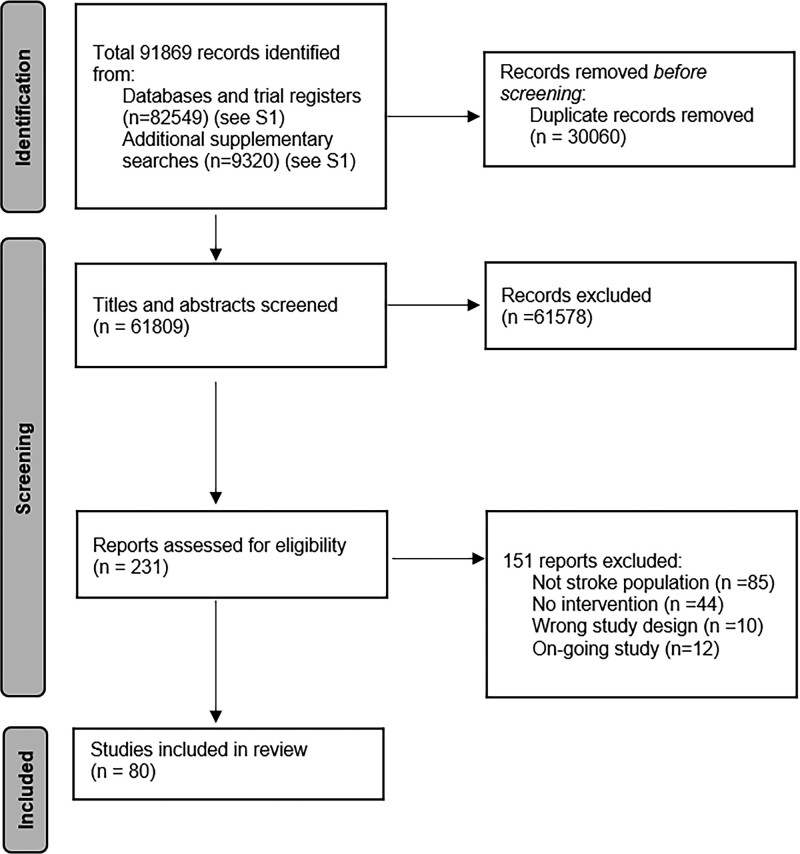
PRISMA (Preferred Reporting Items for Systematic Reviews and Meta-Analyses) diagram for scoping review literature identification.

Of 91 869 titles identified, 80 (893 participants; 869 poststroke perceptual disorders) met our inclusion criteria (Figure 1; Table S2). Interventions and participants were summarized by perceptual disorder: vision (Table 1), somatosensation (Table 2), and other sensory domains (Table 3).

**Table 1.**
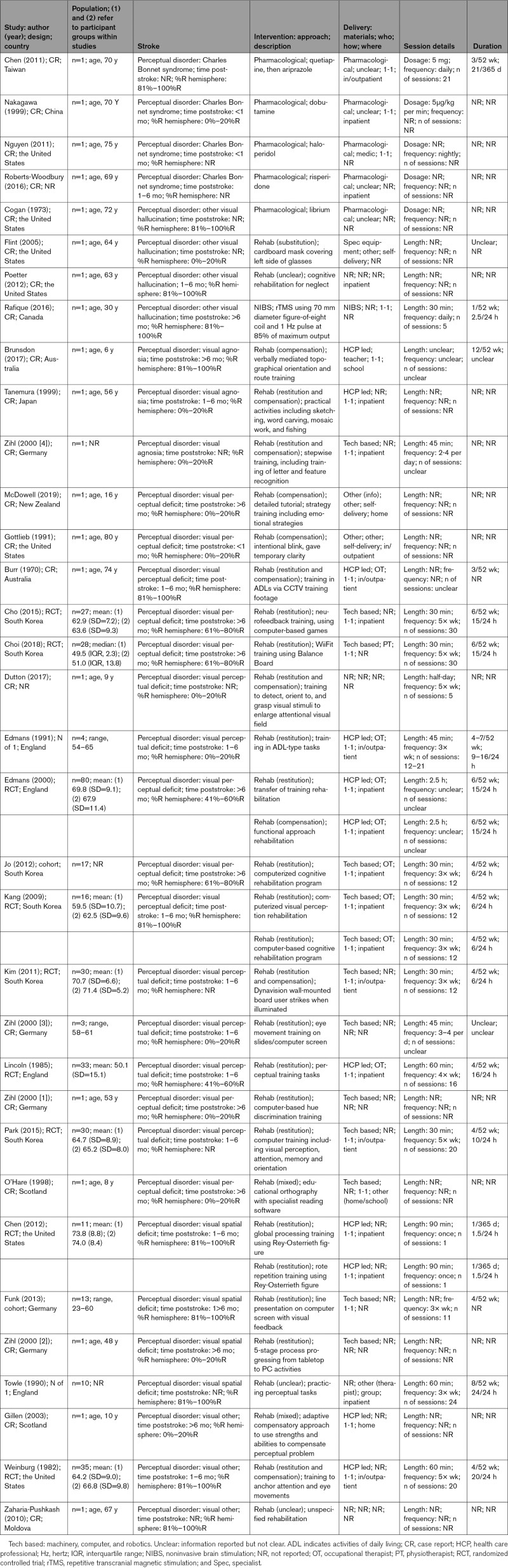
Visual Perceptual Disorders: Details of Studies, Population, and Interventions

**Table 2.**
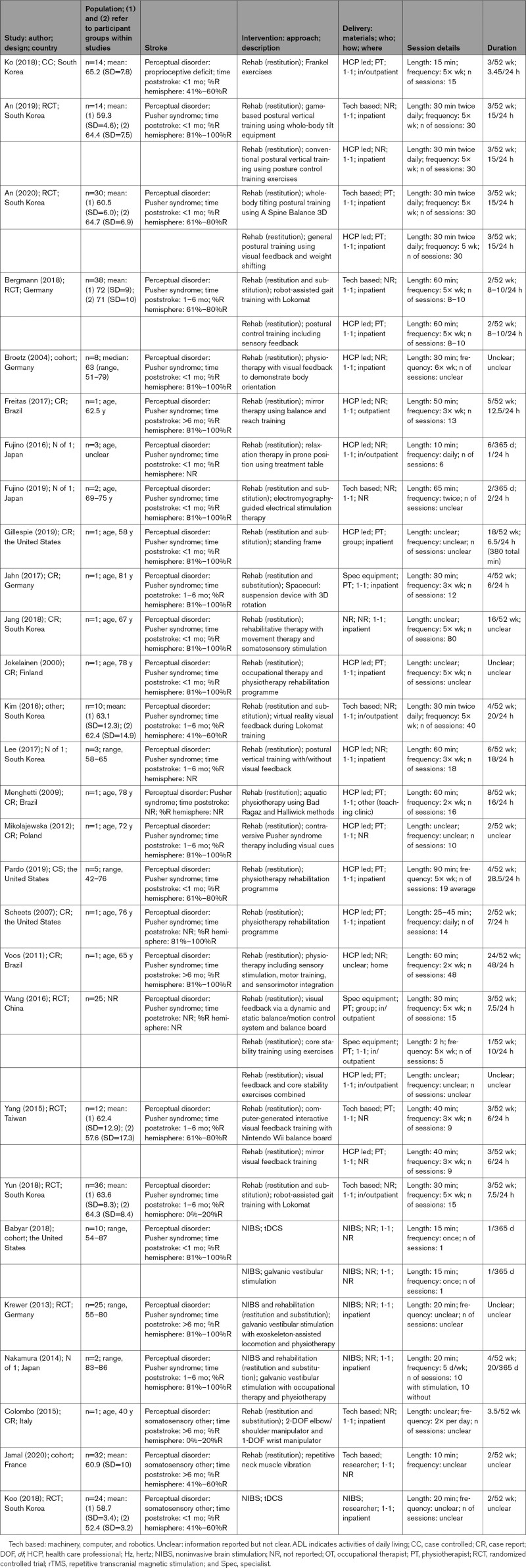
Somatosensation Perceptual Disorders: Details of Studies, Population, and Interventions

**Table 3.**
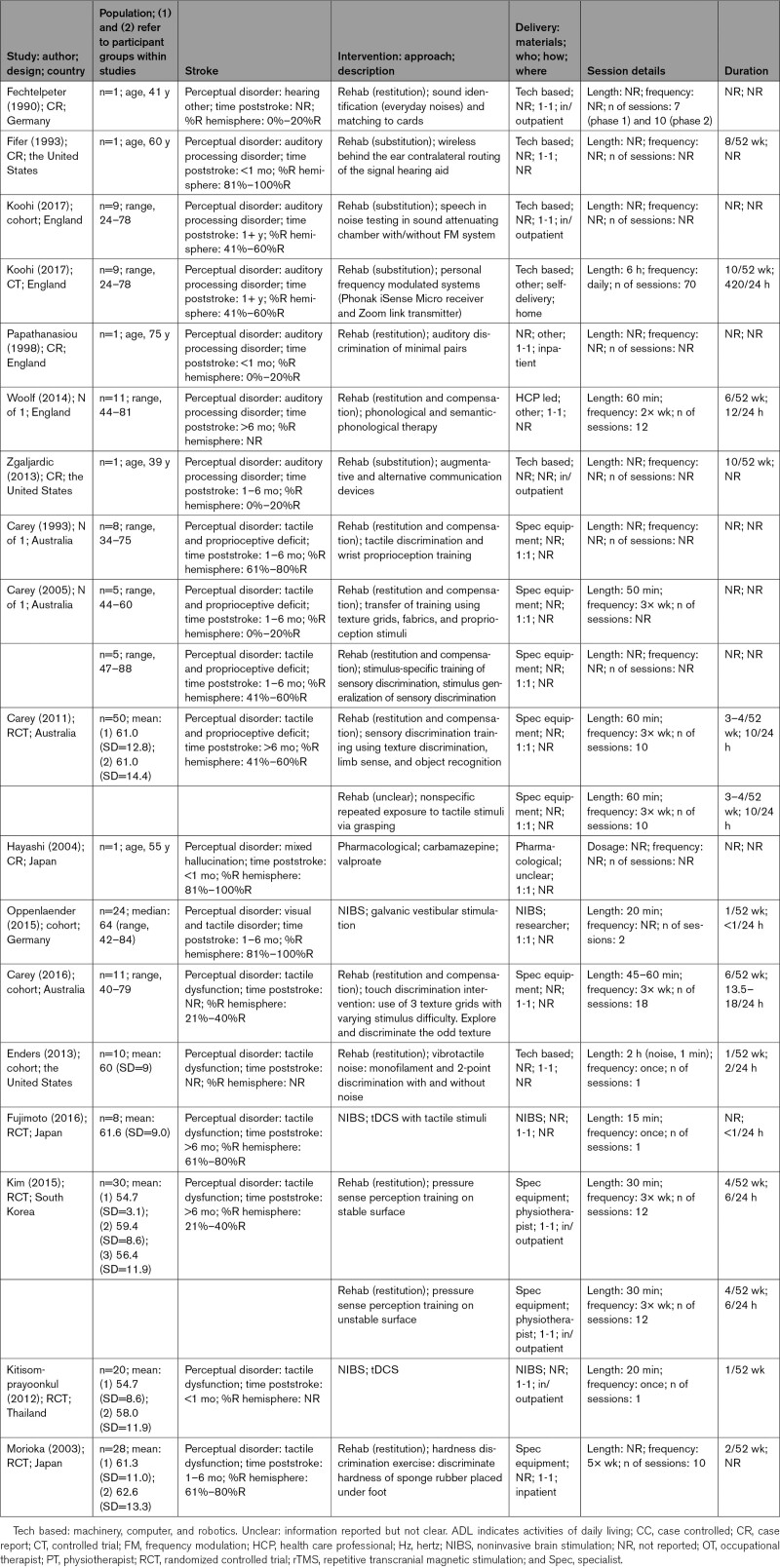
Hearing, Touch, and Mixed Perceptual Disorders: Details of Studies, Population, and Interventions

### Included Studies

**Figure 2.**
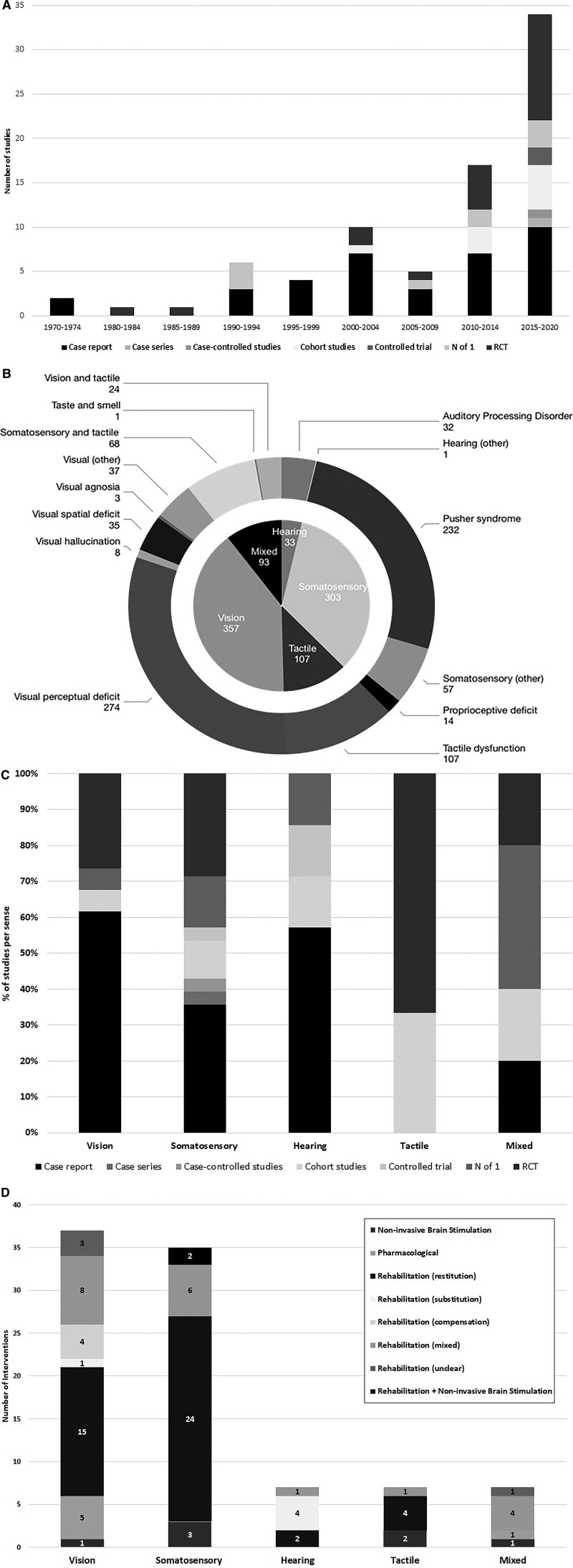
**Study and participant data. A**, The number of different study designs used, by year of publication. **B**, The total number of participants included in studies for each sense (inner ring) and each perceptual disorder (outer ring). **C**, The percentage of different study designs used for each sense. **D**, The number of interventions using a specific intervention approach, for each sense. *The categorization of perceptual deficit signified that study participants had a range of different perceptual issues or who were diagnosed using a test that did not specify the nature of the disorder.

Case reports (36/80; 45.0%) and randomized controlled trials (RCTs; 22/80; 27.5%) were common, with RCTs accounting for most participants (630/893; 70.5%). Study sample sizes ranged from 1 to 80 participants (median, 3.5; interquartile range, 1–16.5). Most were based in Asia (27/80; 33.8%) or Europe (26/80; 32.5%). Study numbers are increasing with time (Figure 2A); of RCTs, 54.5% (12/22) were conducted 2015 to 2020. Involvement of stakeholders, such as stroke survivors, carers, or clinicians, in the research development and delivery (as opposed to as participants) was not reported in any included studies.

Time point poststroke ranged between <1 month (19/80; 23.8%), 1 to 6 months (25/80; 31.3%), and >6 months (20/80; 25.0%). Right hemisphere lesions were common (39/80 studies [48.8%] recruited >60% participants with right-sided lesions), and the mean proportion of women was 34.8% (SD, 33.8). Stroke severity was rarely reported (13/80; 16.3%). Young people (<18 year olds) were represented by 5 single case reports, all describing visual perceptual disorders; the remaining study participants were most commonly aged 18 to 65 years (43/80; 53.8%; Table S3).

### Nature of Perceptual Disorder

Vision (34/80; 42.5% studies; n=357/893; 40.0%) and somatosensation disorders (28/80; 35.0% studies; n=303/893; 33.9%) were most frequently reported. Common disorders included Pusher syndrome^37^ (24 studies), visual perceptual deficits (16 studies), and visual hallucination (8 studies; Figure 2B; disorder definitions in Table S4).

There was variation in study designs addressing each sense (Figure 2C). Some clusters emerged, with specific designs and interventions for specific perceptual disorders; 5 pharmacological intervention case studies examined visual/audiovisual hallucinations; 6 RCTs addressed Pusher syndrome rehabilitation interventions.

### Interventions

Ninety-three perceptual disorder interventions were described across 80 studies (Table S5). Rehabilitation interventions were common (78/93; 83.9%) and primarily restitutive in nature (45/93; 48.4%). Other interventions included NIBS (7/93; 7.5%) and pharmacological interventions (6/93; 6.5%; Figure 2D). Surgical and assessment-based interventions were absent.

#### Overview of the Interventions and Intervention Provider

Interventions often involved therapeutic input from a health care practitioner (HCP; 30/93; 32.3%), such as training and support during specific physical activities, rather than physical materials. Technology-based (robotics or computer) tasks were common (28/93; 30.1%), followed by other specialist tools (13/93; 14.0%; eg, training blocks of different colors and sizes).

Descriptions of intervention delivery procedures (55/93; 59.1%) and providers (54/93; 58.1%) were limited or unclear. Where reported, interventions were predominantly delivered in hospital (56/93; 60.2%) on a one-to-one basis (76/93; 81.7%) lasting ≤1 month (42/93; 45.2%). Three (3.2%) were delivered within a participant’s home. Few interventions lasted >3 months (4.3%; 4/93).

#### Interventions for Individual Sensory Domains

Visual perception disorder interventions used the widest range of approaches: rehabilitation (restitution, 15/37; mixed, 8/37) and pharmacological (5/37; Table 1). Restitution interventions used technology (10/15), often interactive computer-based training of visual skills, while HCP-led interventions taught compensatory skills in real-world simulation tasks. Pharmacological interventions exclusively addressed hallucinations but were solely reported in case reports, with limited details. Vision studies were lacking information on who delivered interventions, where, and for how long.

Four somatosensory disorder intervention approaches were used: rehabilitation (restitution, 24/35; mixed, 6/35), NIBS (3/35), and rehabilitation+NIBS (2/35; Table 2). Most often, interventions were HCP led (17/35), involving physical activities to retrain postural control, with technology-based interventions (9/35) often providing robot-assisted gait training. Interventions were predominantly delivered on a one-to-one basis (32/35), in an inpatient setting (18/35), for ≤1 month (25/35).

Hearing perception disorder intervention reports all describe rehabilitation approaches, primarily technology based (hearing aids; 5/7). Tactile perception disorder interventions (n=7) involved rehabilitation (n=5) or NIBS (n=2). HCPs were less involved in interventions for this disorder, using technology (n=1/5; vibrotactile stimulation) or equipment (n=4/5; tasks with different textures or hardness).

We identified no interventions targeting individual smell or taste perceptual disorders.

### Outcomes Measured

The most frequently measured outcomes were perceptual function (60/80; 75.0%), motor/sensorimotor (32/80; 40%), activities of daily living (18/80; 22.5%), and sensory outcomes (12/80; 15%; Table 4). Outcomes were captured immediately (31/80; 38.8%), ≤1 month (9/80; 11.3%), 1 to 3 months (9/80; 11.3%), and >3 months (12/80; 15.0%) after intervention.

**Table 4.**
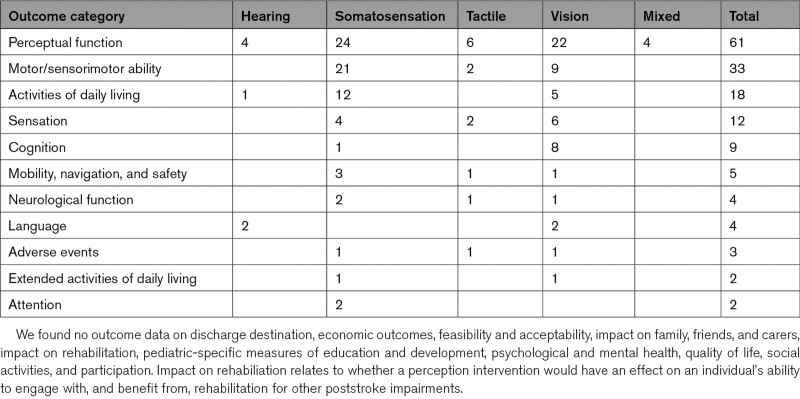
Outcome Measure Domains Reported

Verbatim summaries of study findings are given in Table S6.

## Discussion

### Summary of Findings

Our scoping review scoped the breadth and nature of perceptual disorder interventions poststroke, highlighting gaps in the evidence base. We identified 80 studies that explored predominantly visual or somatosensory perceptual problems, typically using a case report design. Interventions were frequently rehabilitative, with an approach that targeted improvements in the impaired function. Interventions most often involved direct training by an HCP, as well as those using technology-based devices and specialist equipment. Interventions reported were typically hospital based, lasting up to 4 weeks. Few captured outcomes beyond initial postintervention training effects. Perceptual and motor/sensorimotor skills were the most common outcomes reported. We noted an encouraging upward trajectory in the number of perceptual disorder (especially somatosensation) research reports, particularly RCT designs, since 2015.

### Gaps in the Evidence

Our scoping review reveals the paucity of evidence informing perceptual disorder interventions poststroke. Key gaps include lack of research addressing perceptual disorders in pediatric populations; interventions for stroke-related hearing, taste, touch, and smell disorders; RCTs; and stakeholder-informed research. While a range of study designs are needed in early-stage intervention development, high-quality RCTs are important in building the evidence base related to treatment effectiveness. Of the 80 studies in this review, only 22 were RCTs; this is significantly lower than, for example, the 65 current trials in neglect found in a recent review.^38^

#### Perception Terminology

The range and complexity of perceptual terminology continues to be a challenge.^25^ Despite achieving agreement on our definitions and included senses/disorders, we encountered challenges applying our a priori inclusion and exclusion criteria. Perception was inadequately reported, thus determining whether disorders affected perception, cognition, sensation, attention, or a mix of these was difficult. Inconsistent terms for similar conditions across pediatric and adult populations, and across senses, plus use of complex, Latinate terminology hindered transparency and clarity of interpretation. Clear statements of the nature of disorders, and how intervention rationale or mechanisms relate to perception, are needed. International, multidisciplinary consensus on the terminology used would serve to progress the field of research and improve awareness, multidisciplinary identification, and intervention for those affected.

#### Pediatric Perceptual Disorders

Five pediatric case reports on stroke survivors with visual perceptual problems were identified; this is in keeping with the extremely limited evidence base for pediatric stroke clinicians.^39^ Some additional studies that addressed visual perception or cerebral/cortical visual impairment were identified, but as it was unclear whether impairments were stroke related, they were excluded from our review. Demographic information for both neonatal and later childhood perceptual impairment studies is required to support transparency, interpretation, and implementation of emerging research findings.

The limited intervention research involving pediatric stroke populations may reflect the conflicting evidence about the nature, extent,^40^ and expectations of recovery due to neurodevelopmental plasticity.^41^ Evidence of perceptual deficit persistence, and factors associated with pediatric resilience and recovery across sensory modalities, needs greater prominence.

#### Lack of Studies of Hearing, Taste, Touch, and Smell

Interventions for perceptual disorders relating to hearing, taste, touch, and smell were rarely identified; this may reflect stroke survivors’ limited access to specialists, training, and consequently limited awareness of the frequency and impact of these disorders. Assessment and management of visual and somatosensory disorders are more established components of poststroke rehabilitation, giving the impetus to provide evidence to underpin care.^15^ Establishing evidence of the prevalence, presentation, recovery, and impact of hearing, taste, touch, and smell perception disorders after stroke is required to inform clinical care and further research in this field.

#### High Proportion of Case Report Designs

Case reports and RCTs were the most frequent study designs included in this scoping review. Case reports described personalized interventions to individuals with multifaceted perceptual disorders, making their clinical relevance and representativeness difficult to establish. The recent growth in RCT reports is welcome and in keeping with other areas of stroke rehabilitation.^42^ Trial participant numbers were low, however, raising questions about sufficient statistical power to determine clinical and cost-effectiveness. Inadequate reporting of treatment feasibility, fidelity, and outcome measures in RCTs and a lack of cohort and n-of-1 studies were evident. The use of a structured development process for perceptual disorder interventions would support exploration of mechanisms of action, dosage, and target group, informing the development and conduct of RCTs.^43,44^

#### Limited Involvement of Stakeholders

No included study reported the involvement of stroke survivors, carers, clinicians, or other stakeholders in the study design or conduct (as opposed to as participants). Similarly, we identified no qualitative studies exploring experiences of stroke survivors, carers, or clinicians. Other areas of concern were a lack of real-world, community-based studies; feasibility or economic outcomes; follow-up post-initial posttreatment evaluations; and outcomes capturing transfer of intervention effects to daily life. The benefits of stakeholder involvement are well recognized^45^ and would enhance the relevance, implementation, and impact of future research.

### Strengths and Limitations

Our scoping review used a broad and rigorous search of electronic databases and Grey literature, adopting a comprehensive definition of perceptual disorders. Despite these efforts, due to the complex nature of the topic and terminology, some relevant articles may have been missed. Our multidisciplinary clinician-research team had expertise in review methodologies, stakeholder involvement, stroke rehabilitation, cognitive disorders, psychology (adult and pediatric), and hearing, taste, smell, somatosensory, and vision disorders. In addition, involvement of our stakeholder group maximized the relevance and accessibility of our findings. In the absence of a universally accepted intervention categorization, we utilized an existing method to support categorization consistency, relevant to perceptual disorder research^34^ but which may not necessarily directly align with other categorization approaches.^44^ As a scoping review, we did not conduct quality appraisal, and thus comment on quality or generalizability of study findings was not possible.

### Conclusions

Our review provides a comprehensive overview of the evidence relating to interventions for perceptual disorders following adult and childhood stroke. Interventions are under-researched, and the terminology used is a barrier to understanding. Key evidence gaps include interventions for pediatric populations, and for stroke-related hearing, taste, touch and smell perception disorders. Rigorous study design, conduct, and reporting, incorporating fuller involvement of stroke survivors, carers, and clinicians, is needed to address perceptual disorders after stroke.

## Article Information

### Acknowledgments

We would like to acknowledge the invaluable contribution of our clinical and lived-experience stakeholders. This included Graham Esson, Prof Carl Philpott, Dr Gera de Haan, Dr Christine Johnson, and Dr Kathleen Vancleef. We would like to thank Dr Julie Duncan Miller for her assistance in screening studies.

### Sources of Funding

This project is funded by the National Institute for Health Research (NIHR; NIHR Health Technology Assessment [NIHR 128829]) and will be published in full in the NIHR Journals Library. Further information is available at https://fundingawards.nihr.ac.uk/award/NIHR128829. This report presents independent research commissioned by the NIHR. Dr Hazelton is funded by the Stroke Association (TSA), UK (SA L-NC 20\100003); NMAHP RU and Dr Brady is funded by the Chief Scientist Office (CSO), Health and Social Care Directorates, United Kingdom. The views and opinions expressed by authors in this publication are those of the authors and do not necessarily reflect those of the National Health Service (NHS), the NIHR, Medical Research Council, Clinical Commissioning Facility, NIHR Evaluation, Trials and Studies Coordinating Centre (NETSCC), TSA, CSO, the NIHR Health Technology Assessment (NIHR 128829) program or the Department of Health, United Kingdom.

### Disclosures

Dr Brady has disclosed grants from the National Institute for Health Research Health Services and Delivery Research UK including the Health Technology Assessment Programme, Glasgow Caledonian University studentships, and grants from the Tavistock Trust for Aphasia. Dr Nicolson has disclosed a contract from the Metix Medical and conference expenses from the Association for Borderlands Studies World Conference. Dr Hazelton has disclosed funding from the Stroke Association (lectureship SA L-NC 20\100003). The other authors report no conflicts.

### Supplemental Material

Supplemental Methods S1–S3

Tables S1–S6

## Supplementary Material


