# Supplementary material for: Perceptual Disorders After Stroke: A Scoping Review of Interventions
Source: Stroke. 2022 Mar 18;53(5):1772–87. doi: 10.1161/STROKEAHA.121.035671 (PMC9022686; doi:10.1161/STROKEAHA.121.035671)
Supplement: Supplementary file 1 [file str-53-1772-s001.pdf]

SUPPLEMENTAL MATERIAL

PIONEER
Perceptual disorders after stroke intervention evidence review

List of Supplemental material

Supplemental Methods S1: Review Methods

Supplemental Methods S2: GRIPP2 summary of stakeholder involvement

Supplemental Methods S3: PRISMA-ScR Checklist

Supplemental Table S1: Data extraction variables and categorization applied

Supplemental Table S2: Included studies

Supplemental Table S3: Characteristics of included studies, and participant demographics

Supplemental Table S4: Definitions of perceptual disorders

Supplemental Table S5 Summary of intervention descriptions

Supplemental Table S6: Findings from each study

Supplemental Methods

Methods S1: Review Methods

Protocol and registration information

Our scoping review protocol is registered with PROSPERO (CRD42019160270)*

Information sources

Electronic bibliographic databases and clinical trial registers searched during (07/02/2020) included:

- Cochrane Stroke Group's Register, the Cochrane Central Register of Controlled Trials (CENTRAL), and the Cochrane Database of Systematic Reviews (CDSR) in the Cochrane Library (2020, Issue 1);
- MEDLINE (Ovid; from 1966);
- EMBASE (Ovid; from 1980);
- ERIC (EBSCO; from 1966);
- CINAHL (EBSCO) (Cumulative Index to Nursing and Allied Health Literature; from 1982)
- AMED (Ovid; from 1885);
- PsycINFO (Ovid; from 1806);
- Epistemonikos Database (https://www.epistemonikos.org/);
- Web of Science – Core Collection (Indexes= Science Citation Index Expanded (SCI-EXPANDED), Social Sciences Citation Index (SSCI), Arts & Humanities Citation Index (A&HCI), Conference Proceedings Citation Index- Science (CPCI-S), Conference Proceedings Citation Index- Social Science & Humanities (CPCI-SSH) , Book Citation Index– Science (BKCI-S), Book Citation Index– Social Sciences & Humanities (BKCI-SSH), Emerging Sources Citation Index (ESCI) (Timespan=All years);
- Centre for Reviews and Dissemination (Database of Abstracts of Reviews of Effects (DARE) and the NHS Economic Evaluation Database (NHS EED), and Health Technology Assessment (HTA) (https://www.crd.york.ac.uk/CRDWeb/);
- ProQuest Dissertations and Theses Global (from 1997);
- Registers of ongoing trials (US National Institutes of Health Ongoing Trials Register ClinicalTrials.gov (www.clinicaltrials.gov) and World Health Organization International Clinical Trials Registry Platform (apps.who.int/trialsearch).

Supplementary searches included OpenGrey, Grey Matters, Google Scholar (https://scholar.google.com/) and NIHR Clinical Research Network (https://www.nihr.ac.uk/explore-nihr/support/clinical-research-network), the Physiotherapy Evidence Database (PEDro), OTseeker and the PROSPERO International prospective register of systematic reviews.

In addition, national/international guidelines, government websites, relevant healthcare professional (HCP) professional websites and websites of relevant charities and patient support organisations were searched. Research, professional associations or foundations, and experts in the field were also contacted.

Forward citation tracking using Science Citation index and Google Scholar (https://scholar.google.com/) and was completed together with searching reference lists of included studies.

Search Terms - Medline search strategy

1. cerebrovascular disorders/ or basal ganglia cerebrovascular disease/ or exp brain ischemia/ or exp carotid artery diseases/ or exp cerebral small vessel diseases/ or exp intracranial arterial diseases/ or exp "intracranial embolism and thrombosis"/ or exp intracranial hemorrhages/ or stroke/ or exp brain infarction/ or stroke, lacunar/ or vasospasm, intracranial/ or vertebral artery dissection/ or carotid stenosis/ or exp carotid artery injuries/ or intracranial arterial diseases/ or cerebral arterial diseases/ or infarction, anterior cerebral artery/ or infarction, middle cerebral artery/ or infarction, posterior cerebral artery/ or exp carotid arteries/ or endarterectomy, carotid/
2. (stroke$ or poststroke or apoplex$ or cerebral vasc$ or brain vasc$ or cerebrovasc$ or cva$ or SAH).ti,ab
3. ((brain$ or cerebr$ or cerebell$ or vertebrobasil$ or hemispher$ or intracran$ or intracerebral or infratentorial or supratentorial or middle cerebral arter$ or MCA$ or anterior circulation or posterior circulation or basilar arter$ or vertebral arter$ or space-occupying) adj5 (isch?emi$ or infarct$ or thrombo$ or emboli$ or occlus$ or hypoxi$)).ti,ab
4. ((brain$ or cerebr$ or cerebell$ or intracerebral or intracran$ or parenchymal or intraparenchymal or intraventricular or infratentorial or supratentorial or basal gangli$ or putaminal or putamen or posterior fossa or hemispher$ or subarachnoid) adj5 (h?emorrhag$ or h?ematoma$ or bleed$)).ti,ab
5. or/1-4
6. exp perceptual disorders/ or exp perception/
7. hearing disorders/ or hearing loss/ or deafness/ or hearing loss, central/ or hearing loss, sudden/ or hyperacusis/ or olfaction disorders/ or exp somatosensory disorders/ or exp taste disorders/ or vision disorders/ or alice in wonderland syndrome/ or amblyopia/ or blindness/ or blindness, cortical/ or color vision defects/ or diplopia/ or hemianopsia/ or photophobia/ or scotoma/ or vision, low/
8. (percept$ adj3 (impair$ or problem$ or abilit$ or deficit$ or distortion$ or defect$ or disabilit$ or disturbance$ or disorder$ or discriminat$ or deaf$)).ti,ab
9. (agnosis or agnosia or prosopagnosia or prosophthalmia or Alice in Wonderland syndrome or Todd syndrome or all?esthesia$ or syn?esthesia$ or hypoesthesia or hyperesthesia).ti,ab
10. sensation/ or hearing/ or smell/ or taste/ or touch/ or vision, ocular/ or color vision/ or exp mesopic vision/ or night vision/
11. (somatosensory$ or (sensor$ adj3 (input$ or stimul$ or deficit$ or distortion$ or defect$ or disabilit$ or disturbance$ or disorder$ or discriminat$ or processing or percept$ or hallucination$ or feedback or discriminat$ or dysfunction$ or recogn$ or interpretation)) or somatosognosia or asomatognosia or somatoparaphrenia or (body adj3 (schema or orientation))).ti,ab
12. exp Proprioception/

13. (propriocep$ or (kin?esthetic adj3 (percept$ or discriminat$))).ti,ab
14. ((odo?r$ or smell$ or olfact$ or scent$ or aroma or flavo?r) adj3 (memory or acuity or function$ or percept$ or perceive$ or discriminat$ or distinguish$ or recept$ or sensitiv$ or hedonics or deprivation or hallucinat$)).ti,ab
15. (anosmia or anodmia or anosmy or Kallmann syndrome or dysosmia or hyposmia or hyposphresia or phantosmia or par?osmia or ageusia or hypogeusia or dysgeusia or troposmia or euosmia or cacosmia or malodour or superosmia).ti,ab
16. (ageusia or dysgeusia or parageusia or phantogeusia or hypogeusia or amblygeustia or hypogeusesthesia or hyp?esthesia or superosmia or phantosmia or parosmia or troposmia or euosmia or cacosmia or dysosmia or hypergeusia or phantogeusia or hyperosmia or hyposmia).ti,ab
17. ((gustat$ or tast$) adj3 (acuity or percept$ or perceive$ or discriminat$ or distinguish$ or recept$ or sensitiv$ or hallucination$ or abnormalit$ or distortion$ or disturbance$ or anomal$ or loss or an?esthesia or absence or phantom)).ti,ab
18. (((speech or speak$ or voice or spoken or acoustic or audio or auditory or sound or pitch or prosody or binaural or phoneme) adj3 (percept$ or processing or stimul$ or distinguish$ or discriminat$)) or hyperacusis or misophonia or phonophobia or sonophobia or amusia or King Kopetsky syndrome).ti,ab
19. (amblyop? or aniseikonia or oscillopsia or xanthopsia or d?plop$ or polyop$ or metamorphopsia or m?cropsia or ((vision or visual or visual?percept$ or visuo?spatial or visuo?construct$ or ocular or optokinetic or optic$ or oculomotor spatial) adj3 (illusion or blurry or overload or double or percept$ or perceive$ or discriminat$ or distinguish$ or recept$ or sensitiv$ or hallucination$ or abnormalit$ or distortion$ or disturbance$ or anomal$ or disorientation or allachethesia or deficit$ or defect$ or disabilit$ or disorder$ or processing or dysfunction$ or recogn$ or interpretation or analysis or comprehension)) or stereoillusion or kakopsia or kalopsia or pelopsia or archromatopsia or akinetopsia or telopsia or stereopsis or palinopsia or teleopsia or simultanagnosia).ti,ab
20. (entomopia or palinopsia or asteropsis or strabismus or Anton syndrome or Balint syndrome or blindsight or achromatopsia or hyperchromatosis or ((facial or face) adj3 intermetamorphosis) or (visual adj3 anoneria)).ti,ab
21. ((figure or shape or orientation or form or colo?r or textur$ or crowding or contour or object or face or faces) adj3 recogn$).ti,ab
22. (astereognosia or stereognosis or astereognosis or paraesthesia or hypersensitivity or ((tactile or haptic$ or touch) adj3 (stimul$ or memory or acuity or sens$ or percept$ or processing or stimul$ or distinguish$ or discriminat$ or anisotropy or locali?ation))).ti,ab
23. or/6-22
24. 5 and 23

Study selection

Study selection processes implemented were in line with scoping review guidance, using the following criteria:

Participants: of any age (adults and children) with stroke-related disorders of perception

Studies which combine stroke and non-stroke populations were included and coded to indicate whether they are a stroke-only or mixed population.

Studies that combine perceptual disorders with sensory or cognitive disorders, or where the precise nature of the disorder could be determined were included and coded to indicate this.

Intervention: all interventions that expressly address a perceptual disorder. We envisioned these may include rehabilitation, pharmacological, screening/ assessment interventions and possibly surgical. We included and coded all interventions that addressed perceptual disorders across more than one sense.

Study Design: all quantitative, qualitative and mixed-methods primary research studies, exploring clinical effectiveness, economic outcomes or implementation of interventions.

Setting: all settings, including hospital, community and out-patients, and any geographical location

Date and Language: All published and unpublished studies in any language, with no date limitations

Titles were assessed for eligibility by one reviewer (KM) with remaining abstracts and subsequent full texts evaluated by two reviewers (KM, CH). Third reviewers were consulted with areas of uncertainty or disagreement.

*In our original protocol, we had planned to search the following databases: Applied Social Sciences Index and Abstracts (ASSIA), PsycLIT database and Latin American and Carribean Health Services Literature (LILACS). Following the inception meeting with key stakeholders to develop the search strategy, it was clear that there was potentially significant overlap across these databases and in line with the publication of Bramer WM, Rethlefson ML, Kleijnen J, Franco OH (2017) Optimal database combinations for literature searches in systematic reviews: a prospective exploratory study. Systematic Reviews, 6, 245, we decided to include the Web of Science Core Collection instead.

Definition of perception

Our definitions of perception, and how to apply these, were developed and agreed through a full-day consensus process at the start of our project. The meeting was attended by the researcher team, and the stakeholder group of stroke survivors, carers and specialist clinicians in the field of perception. Each term was discussed in depth, definitions co-produced, and confirmed through a voting process.

The definition of perception agreed was: "**specific mental functions of recognizing and interpreting sensory stimuli".**

The included senses, and their agreed definitions were:

Vision<br><br>(visual)	Processing and understanding visual (vision) information.<br><br>This may include the mental functions of being able to distinguish, discriminate, recognise and interpret visual information.
Hearing<br><br>(auditory)	Processing and understanding auditory (hearing) information.<br><br>This may include the mental functions of being able to distinguish, discriminate, recognise and interpret auditory information.
Taste<br><br>(gustatory)	Processing and understanding gustatory (taste) information.<br><br>This may include the mental functions of being able to distinguish, discriminate, recognise and interpret gustatory information.
Smell<br><br>(olfactory)	Processing and understanding olfactory (smell) information.<br><br>This may include the mental functions of being able to distinguish, discriminate, recognise and interpret olfactory information
Touch	Processing and understanding tactile information*. This may include the mental functions of being able to distinguish, discriminate, recognise and interpret tactile information.<br><br>*Tactile is described as "understanding information from the skin"
Somatosensation<br><br>(including proprioception)	Processing and understanding somatosensory information. This may include the mental functions of being able to distinguish, discriminate, recognise, and interpret somatosensory information.

NB somatosensation and touch were treated separately. Although tactile sensation can be considered a component of somatosensation, it was the decision of the group (after discussion) to present these individually, as they were considered separate – and distinguishable - functions. It also was felt this would aid understanding by stroke survivors and carers, for whom touch is well-recognised as one of the five "traditional" senses.

We did not include studies related to pain.

Application of definitions within the review process

To apply our eligibility criteria two independent reviewers considered whether a disorder was perceptual, based on a study's description of the function/impairment being studied, and our agreed definitions. A third a relevant topic expert was consulted where necessary: for somatosensory and tactile disorders (where decision making was often found to be difficult), a stroke-specialist physiotherapist was involved in decision-making.

Participant populations with complex disorders (including disorders of balance and Pusher Syndrome) proved challenging. A third topic expert was involved in all decisions, which were pragmatic, but considering relevant theory. Studies focused on balance were excluded, as balance was considered to (i) frequently incorporate a broad range of non-perceptual input, (ii) be a stage 'after' perception (analogous to reading and vision) and (iii) has an evidence base that often takes a physical function approach, and whose inclusion could make the results less meaningful. Studies of Pusher Syndrome, defined as "a clinical disorder following left or right brain damage in which patients actively push away from the non-hemiparetic side, leading to a loss of postural balance"[37] were included. Whilst the aetilogy and mechanism of the disorder are not fully understood, it was fundamentally as disorder of perception, where "perception of body posture in relation to gravity is altered" [37] and thus eligible for inclusion.

Disorders purely of sensation, such as visual field loss, and those purely of attention, such as visual neglect, were not included, as they were fell outside the definition of perception used in this review.

Methods S2: GRIPP2 summary of stakeholder involvement

GRIPP2 short form

<table>
<tr><th>Section and topic</th><th>Item</th><th>Reported on page No</th></tr>
<tr><td>1: Aim</td><td>Report the aim of PPI in the study
The key aim of PPI was to maximise the quality, relevance and accessibility of our work. Specific aims included:
Agree definitions on key terms of relevance to the review (including "perception" and related terms)
Agree, and prioritise, outcomes of relevance to the review
Contribute to decisions on inclusion / exclusion of papers
Contribute to interpretation of findings
Support dissemination</td><td>In Methods + additional information here</td></tr>
<tr><td>2: Methods</td><td>Provide a clear description of the methods used for PPI in the study
We had three key PPI strategies:
CO-PRODUCTION. Author DJN (a stroke survivor with a related perceptual problem) was a co-applicant on the funding application, and has a shared responsibility for this research. DJN has contributed to decisions around the design, conduct, interpretation and reporting of this research, and taken a lead role in the involvement of the Lived Experience Group (see below).
LIVED EXPERIENCE GROUP. This group comprises 5 individuals (including 2 stroke survivors, 2 carers of stroke survivors, and 1 parent of a child with perceptual problems after stroke).
CLINICAL EXPERT GROUP. This group comprises 4 clinicians with expertise relating to different areas of perception (vision, hearing, smell/taste).
Led by DJN, and our evidence synthesis PPI expert (AP), these groups had one face-to-face meeting at the start of the project, and then had four video-conference meetings, plus additional communication via email. Methods used within meetings comprised facilitated discussion, informed by participatory methods, to ensure all opinions were heard and represented. We used the nominal group technique, and experience of James Lind Alliance consensus methods, to reach consensus on key points, as this provides a structured format which is democratic, fosters equal participation and can facilitate generation of new ideas. Key stages to reach consensus involved (i) generation of ideas, (ii) sharing ideas, (iii) group discussion, (iv) voting and ranking.</td><td>In Methods + additional information here</td></tr>
<tr><td>3: Study results</td><td>Outcomes—Report the results of PPI in the study, including both positive and negative outcomes
Definitions were agreed and outcomes identified and prioritised, and are</td><td>Reported here. Manuscript</td></tr>
</table>

Section and topic	Item	Reported on page No
	reported within this paper.<br><br>After each meeting all PPI contributors completed a record of involvement. This record captures each member's perception of their role at that stage (including the degree of control / influence that the felt they had over the review), what they felt they influenced, what was good / not so good, and any additional comments.<br><br>The results of these records of involvement will be fully reported within a separate paper (after completion of all stages of the project, which includes a Cochrane review in addition to this reported scoping review).	reporting the details of this in preparation.
**4: Discussion and conclusions**	**Outcomes—Comment on the extent to which PPI influenced the study overall. Describe positive and negative effects**<br>PPI was central to decision making for this scoping review. The PPI input into this scoping review:<br>• provided clear definitions of key terms which have been used to inform and structure the scoping review<br>• provided a list of important outcomes of interest which informed the data extraction and presentation of results from the scoping review<br>• influenced decisions on the presentation of results within this paper	Reported here. Manuscript reporting the details of this in preparation.
**5: Reflections/ critical perspective**	**Comment critically on the study, reflecting on the things that went well and those that did not, so others can learn from this experience**<br><br>Careful planning at the protocol stage provided us with a clear pre-planned strategy and process for PPI. We have followed our pre-planned methods as fully as possible (moving to more online meetings than anticipated due to COVID restrictions), and successfully met our aims. Our PPI in this study is ongoing, as the PPI groups are continuing to contribute to a subsequent review (Cochrane review) and to other dissemination activities based on the results of our work. We plan to produce a critical reflection, including data from feedback forms provided by all PPI members after each meeting, at the end of this project.	Reported here. Manuscript reporting the details of this in preparation.

PPI=patient and public involvement

Whilst GRIPP uses the term PPI, we used the term stakeholder in this review, to denote the involvement of clinical specialists, as well as stroke survivors and carers

Methods S3: Preferred Reporting Items for Systematic reviews and Meta-Analyses extension for Scoping Reviews (PRISMA-ScR) Checklist

SECTION	ITEM	PRISMA-ScR CHECKLIST ITEM	REPORTED ON PAGE #
**TITLE**			
Title	1	Identify the report as a scoping review.	Title Page
**ABSTRACT**			
Structured summary	2	Provide a structured summary that includes (as applicable): background, objectives, eligibility criteria, sources of evidence, charting methods, results, and conclusions that relate to the review questions and objectives.	1-2
**INTRODUCTION**			
Rationale	3	Describe the rationale for the review in the context of what is already known. Explain why the review questions/objectives lend themselves to a scoping review approach.	4-5
Objectives	4	Provide an explicit statement of the questions and objectives being addressed with reference to their key elements (e.g., population or participants, concepts, and context) or other relevant key elements used to conceptualize the review questions and/or objectives.	4-5
**METHODS**			
Protocol and registration	5	Indicate whether a review protocol exists; state if and where it can be accessed (e.g., a Web address); and if available, provide registration information, including the registration number.	2, 5
Eligibility criteria	6	Specify characteristics of the sources of evidence used as eligibility criteria (e.g., years considered, language, and publication status), and provide a rationale.	5-6
Information sources*	7	Describe all information sources in the search (e.g., databases with dates of coverage and contact with authors to identify additional sources), as well as the date the most recent search was executed.	5-6, Supplementary methods I
Search	8	Present the full electronic search strategy for at least 1 database, including any limits used, such that it could	Supplementary method I

		be repeated.	
Selection of sources of evidence†	9	State the process for selecting sources of evidence (i.e., screening and eligibility) included in the scoping review.	6, Supplementary method I
Data charting process‡	10	Describe the methods of charting data from the included sources of evidence (e.g., calibrated forms or forms that have been tested by the team before their use, and whether data charting was done independently or in duplicate) and any processes for obtaining and confirming data from investigators	6
Data items	11	List and define all variables for which data were sought and any assumptions and simplifications made.	Supplementary table I
Critical appraisal of individual sources of evidence§	12	If done, provide a rationale for conducting a critical appraisal of included sources of evidence; describe the methods used and how this information was used in any data synthesis (if appropriate).	Not done, noted in methods
Synthesis of results	13	Describe the methods of handling and summarising the data that were charted.	7
**RESULTS**			
Selection of sources of evidence	14	Give numbers of sources of evidence screened, assessed for eligibility, and included in the review, with reasons for exclusions at each stage, ideally using a flow diagram.	Figure 1
Characteristics of sources of evidence	15	For each source of evidence, present characteristics for which data were charted and provide the citations.	Citations - Supplementary table II<br>Characteristics - Tables 1,2&3
Critical appraisal within sources of evidence	16	If done, present data on critical appraisal of included sources of evidence (see item 12).	Not done
Results of individual sources of evidence	17	For each included source of evidence, present the relevant data that were charted that relate to the review questions and objectives.	Tables 1-4<br>Supplementary table V
Synthesis of results	18	Summarize and/or present the charting results as they relate to the review questions and objectives.	7-11<br>Figure 2, Supplementary tables

			III, IV
**DISCUSSION**			
Summary of evidence	19	Summarize the main results (including an overview of concepts, themes, and types of evidence available), link to the review questions and objectives, and consider the relevance to key groups.	11
Limitations	20	Discuss the limitations of the scoping review process.	14-15
Conclusions	21	Provide a general interpretation of the results with respect to the review questions and objectives, as well as potential implications and/or next steps.	15
**FUNDING**			
Funding	22	Describe sources of funding for the included sources of evidence, as well as sources of funding for the scoping review. Describe the role of the funders of the scoping review.	15-16

Supplemental Tables

Table S1: Data extraction variables and categorization applied

	Data extracted	Categorization of data: categories applied, definitions
**Study Information**	i. Author<br>ii. Year of publication<br>iii. Type of publication<br>iv. Study title<br>v. Reference<br>vi. Country<br>vii. Author correspondence details	vi. Country – continent category added<br>(Asia, Africa, Europe, N. America, S. America, Australia)
**Study characteristics**	i. Study aims<br>ii. Study design<br>iii. Recruitment method<br>iv. Number of recruitment sites (single vs multiple)<br>v. PPI involvement (yes, not reported)	ii. Study design - categorized (RCT, case report, case series, case-controlled, cross sectional, controlled trial, other)
**Participants**	i. Selection criteria<br>ii. Total study population (n=)<br>iii. Withdrawals (n=)<br>iv. Lost to follow-up (n=)<br>v. Age – measures of age/distribution<br>vi. Adult or child (adult/child)<br>vii. Sex - % female<br>viii. Stroke characteristics<br>ix. Stroke severity<br>x. Type of stroke<br>xi. Hemisphere affected (% right, % left, % bilateral)<br>xii. Presence of other stroke related impairment<br>xiii. Time since stroke<br>xiv. Just stroke survivors (yes, no unclear)<br>xv. Perceptual disorder<br>a. Name<br>b. Sense (hearing, smell, somatosensation, tactile, taste, vision)	v. Age – categorized (Child (<18), Adult (18 - 65), Older adult (>65) adults and older Adult, unclear, not reported)<br>vii. Sex categorized by % female (<25% women,<br>25 - 50% women, 51 - 75% women, >75 % female, unclear, not reported)<br>ix. Stroke severity categorized as if reported (yes/no)<br>x. Type of stroke categorized (ischemic, hemorrhagic, both, unclear, not reported)<br>xi. Hemisphere affected categorized by % right sided (0 - 20% right, 21 - 40% right, 41 - 60 % right, 61 - 80% right, 81 - 100% right, unclear, not reported)<br>xii. Presence of other stroke related impairment categorized (yes single, yes multiple, unclear, not reported)<br>xiii time since stroke categorized (Acute (up to one month), sub-acute (1 to 6 months), Chronic (more than 6 months), unclear, not reported)

	c. Perceptual or sensory-perceptual<br>d. Method of diagnosis (standardized test, self-report, clinical assessment not reported)	
**Interventions**	i. Intervention description<br>ii. Approach<br>iii. Name of intervention<br>iv. n=?<br>v. Rationale reported (yes, partial yes, no*)<br>vi. Materials reported (yes, partial yes, no*), description<br>vii. Procedure reported (yes, partial yes, no*), description<br>viii. Materials access details reported (yes, partial yes, no*)<br>ix. Intervention provider reported (yes, partial yes, no*), description<br>x. Mode of delivery reported (yes, partial yes, no*), description<br>xi. Location of delivery reported (yes, partial yes, no*), description<br>xii. When and how much reported (yes, partial yes, no*, description<br>xiii. Intervention tailored (yes, partial yes, no*)<br>xiv. Was evaluation of adherence or fidelity planned (yes, partial yes, no*)<br>xv. Were any other interventions tested? (yes/no)<br>xvi. Was the intervention delivered alongside usual care (yes, no, unclear)	ii. Approach classified (pharmacological, non-invasive brain stimulation, rehabilitation). Rehabilitation was further classified as restitution (direct training of impaired function), compensation (compensation of deficit by a training/using a spared function) substitution (use of an external device or modification e.g. optic or prosthetic devices, environmental redesign)[1], or a mix of these.<br>Non-invasive brain stimulation includes transcranial magnetic stimulation (rTMS), theta burst stimulation (TBS), and transcranial direct current stimulation (tDCS).<br>vi. Materials categorized (health care professional led, technology (machinery, computer, robotics), brain stimulation, equipment (specialised equipment that does not come under technology), pharmacological, not reported)<br>ix. intervention provider categorised (physiotherapist, occupational therapist, researcher, medical doctor (medic), other, not reported, unclear)<br>x. mode of delivery categorised (one-to-one, self-delivery, group,<br>not reported, unclear)<br>xi. location of delivery categorised (hospital inpatient, hospital outpatient, hospital in- or out-patient, home, not reported)<br>xiii. duration of intervention delivery classified (less than one week, one month or less, between one and three months, more than three months, not reported, unclear)<br>xv. Other intervention type categorised (active, sham, unclear),
**Results**	i. Outcome measures<br>ii. Measurement timepoint<br>iii. Summary of results	i. Outcome measures – categorised based on input for stakeholders (ADL, Perception, EADL, Sensory, Cognitive, Attention, Motor/motor sensory, Mobility, navigation, and safety, Language, QoL, Social activities, skills, and participation, Psychological effects and mental health, Discharge Destination, Adverse events, Economic outcomes, Feasibility outcomes, Compensating for

		perceptual deficits by using other skills, Impact on rehabilitation, Paediatric specific: development and education, Brain (neurological) function, Impact on family, friends, and carers, no outcome measures used)<br>ii. Measurement timepoint - categorized based on longest follow-up timepoint (Immediate (same day as intervention), Medium term (1-3 months), Long term (> 3 months), not reported)

* Partial yes - where some detail is given, but it lacks enough detail for a clinician to be able to deliver the intervention

Table S2: Included studies

No	Study ID	Reference
1	An 2019	An C, Roh J, Kim T, Choi H, Choi K, Gyoung-mo Kim. Visual and somatosensory integration processing is needed to reduce pusher behavior (PB) and improve postural control in hemiplegic patients with acute stroke. *Phys Ther Korea*. 2019;26:57–66.
2	An 2020	An CM, Ko MH, Kim D hyun, Kim GW. Effect of postural training using a whole-body tilt apparatus in subacute stroke patients with lateropulsion: A single-blinded randomized controlled trial. *Ann. Phys. Rehabil. Med.* [Internet]. 2020. https://doi.org/10.1016/j.rehab.2020.05.001
3	Babyar 2018	Babyar S, Santos T, Will-Lemos T, Mazin S, Edwards D, Reding M. Sinusoidal Transcranial Direct Current Versus Galvanic Vestibular Stimulation for Treatment of Lateropulsion Poststroke. *J. Stroke Cerebrovasc. Dis.* [Internet]. 2018;27:3621–3625
4	Bergmann 2018	Bergmann J, Krewer C, Jahn K, Muller F. Robot-assisted gait training to reduce pusher behavior A randomized controlled trial. *Neurology*. 2018;91:E1319–E1327.
5	Broetz 2004	Broetz D, Johannsen L, Karnath HO. Time course of "pusher syndrome" under visual feedback treatment. *Physiother. Res. Int.* 2004;9:138–143.
6	Brunsdon 2007	Brunsdon R, Nickels L, Coltheart M, Joy P. Assessment and treatment of childhood topographical disorientation: A case study. *Neuropsychol. Rehabil.* 2007;17:53–94.
7	Burr 1972	Burr M, Hazen B. The Use of Television in the Rehabilitation of Stroke patients With Perceptual Difficulties. *AOTJ.* 1972;Jan-Mar:19–22
8	Carey 1993	Carey LM, Matyas TA, Oke LE. Sensory loss in stroke patients: Effective training of tactile and proprioceptive discrimination. *Arch. Phys. Med. Rehabil.* 1993;74:602–611.
9	Carey 2005	Carey LM, Matyas TA. Training of somatosensory discrimination after stroke: Facilitation of stimulus generalization. *Am. J. Phys. Med. Rehabil.* 2005;84:428–442.
10	Carey 2011	Carey L, Macdonell R, Matyas TA. SENSe: Study of the effectiveness of neurorehabilitation on sensation: A randomized controlled trial. *Neurorehabil. Neural Repair*. 2011;25:304–313
11	Carey 2016	Carey LM, Abbott DF, Lamp G, Puce A, Seitz RJ, Donnan GA. Same Intervention-Different Reorganization: The Impact of Lesion Location on Training-Facilitated Somatosensory Recovery after Stroke. *Neurorehabil. Neural Repair*. 2016;30:988–1000.
12	Chen 2011	Chen CC, Liu HC. Low-dose aripiprazole resolved complex hallucinations in the left visual field after right occipital infarction (Charles Bonnet syndrome). *Psychogeriatrics*. 2011;11:116–118.
13	Chen 2012	Chen P, Hartman AJ, Priscilla Galarza C, DeLuca J. Global processing training to improve visuospatial memory deficits after right-brain stroke. *Arch. Clin. Neuropsychol.* 2012;27:891–905

14	Cho 2015	Cho HY, Kim K, Lee B, Jung J. The effect of neurofeedback on a brain wave and visual perception in stroke: A randomized control trial. *J. Phys. Ther. Sci.* 2015;27:673–676
15	Choi 2018	Choi D, Choi W, Lee S. Influence of Nintendo Wii Fit Balance Game on Visual Perception, Postural Balance, and Walking in Stroke Survivors: A Pilot Randomized Clinical Trial. *Games Health J.* 2018;7:377–384
16	Cogan 1973	Cogan DG. Visual hallucinations as release phenomena. *Albr. von Graefes Arch. für Klin. und Exp. Ophthalmol.* 1973;188:139–150.
17	Colombo 2016	Colombo R, Sterpi I, Mazzone A, Delconte C, Pisano F. Improving proprioceptive deficits after stroke through robot-assisted training of the upper limb: a pilot case report study. *Neurocase* [Internet]. 2016;22:191–200.
18	Dutton 2017	Dutton GN, Chokron S, Little S, Mcdowell N. Posterior Parietal Visual Dysfunction: An Explanatory Review. *Vis. Dev. Rehabil.* 2017;3:10–22.
19	Edmans 1991	Edmans JA, Lincoln NB. Treatment of Visual Perceptual Deficits after Stroke: Single Case Studies on Four Patients with Right Hemiplegia. *Br. J. Occup. Ther.* 1991;54:139–144.
20	Edmans 2000	Edmans JA, Webster J, Lincoln NB. A comparison of two approaches in the treatment of perceptual problems after stroke. *Clin. Rehabil.* [Internet]. 2000 [cited 2019 May 11];14:230–243
21	Enders 2013	Enders LR, Hur P, Johnson MJ, Seo NJ. Remote vibrotactile noise improves light touch sensation in stroke survivors' fingertips via stochastic resonance. *J. Neuroeng. Rehabil.* 2013;10:1–8.
22	Fechtelpeter 1990	Fechtelpeter A, Goddenhenrich S, Huber W, Springer L. Ansatze Therapie von auditiver Agnosie. *Folia Phoniatr.* 1990;42:83–97.
23	Fifer 1993	Fifer RC. Insular stroke causing unilateral auditory processing disorder: case report. *J. Am. Acad. Audiol.* 1993;4:364–369
24	Flint 2005	Flint AC, Loh JP, Brust JCM. Vivid visual hallucinations from occipital lobe infarction. *Neurology*. 2005;65:754–756
25	Freitas 2017	Freitas ACM, Bezerra LAP, Oliveira PCA de, Freitas LM, Silva SR da, Cirne GN de M, Cacho R de O. Evaluation of the effectiveness of mirror therapy in Pusher Syndrome and hemineglect in post-stroke patients. *Fisioter. Bras.* 2017;18:362–368.
26	Fujimoto 2016	Fujimoto S, Kon N, Otaka Y, Yamaguchi T, Nakayama T, Kondo K, Ragert P, Tanaka S. Transcranial direct current stimulation over the primary and secondary somatosensory cortices transiently improves tactile spatial discrimination in stroke patients. *Front. Neurosci.* 2016;10:1–9
27	Fujino 2016	Fujino Y, Amimoto K, Sugimoto S, Fukata K, Inoue M, Takahashi H, Makita S. Prone positioning reduces severe pushing behavior: Three case studies. *J. Phys. Ther. Sci.* 2016;28:2690–2693.
28	Fujino 2019	Fujino Y, Takahashi H, Fukata K, Inoue M, Shida K, Matsuda T, Makita S, Amimoto K. Electromyography-guided electrical stimulation therapy for patients with pusher behavior: A case series. *NeuroRehabilitation*. 2019;45:537–545
29	Funk 2013	Funk J, Finke K, Reinhart S, Kardinal M, Utz KS, Rosenthal A, Kuhn C, Müller H, Kerkhoff G. Effects of feedback-based visual line-

		orientation discrimination training for visuospatial disorders after stroke. *Neurorehabil. Neural Repair*. 2013;27:142–152
30	Gillen 2003	Gillen J a, Dutton GN. Balint ' s syndrome in a 10-year-old male: A Case report. *Dev. Med. Child Neurol.* 2003;45:349–352.
31	Gillespie 2019	Gillespie J, Callender L, Driver S. Usefulness of a standing frame to improve contraversive pushing in a patient post-stroke in inpatient rehabilitation. *Baylor Univ. Med. Cent. Proc.* [Internet]. 2019;32:440–442.
32	Gottlieb 1991	Gottlieb D, Calvanio R, Levine D. Reappearance of the Visual Percept Afterr Intentional Blinking in a Patient with Balint's Syndrome. *J. Clin. Neuroophthalmol.* 1991;11:62–65.
33	Hayashi 2004	Hayashi R. Olfactory illusions and hallucinations after right temporal hemorrhage. *Eur. Neurol.* 2004;51:240–241.
34	Jahn 2017	Jahn K, Müller F, Koenig E, Krewer C, Tillmann S, Bergmann J. Rehabilitation of verticality perception using a new training method. *J. Neurol.* 2017;264:26–27.
35	Jamal 2017	Jamal K, Leplaideur S, Rousseau C, Chochina L, Moulinet-Raillon A, Senal N, Bonan I. The long-lasting effects of repetitive neck muscle vibration on postural disturbances in standing position in chronic patients, *Neurophysiologie Clinique* 2017;47():341-342
36	Jang 2018	Jang SH, Lee H Do. Recovery of an injured medial lemniscus with concurrent recovery of pusher syndrome in a stroke patient: A case report. *Med. (United States)*. 2018;97:21–23.
37	Jo 2012	Jo K, Yu J, Jung J. Effects of virtual reality-based rehabilitation on upper extremity function and visual perception in stroke patients: A randomized control trial. *J. Phys. Ther. Sci.* 2012;24:1205–1208
38	Jokelainen 2000	Jokelainen L, Jokelainen M. Työntöoireyhtymä. *Duodecim*. 2000;116:144–7.
39	Kang 2009	Kang SH, Kim DK, Seo KM, Choi KN, Yoo JY, Sung SY, Park HJ. A computerized visual perception rehabilitation programme with interactive computer interface using motion tracking technology - A randomized controlled, single-blinded, pilot clinical trial study. *Clin. Rehabil.* 2009;23:434–444
40	Kim 2011	Kim EJ, Lee KE, Lee KL, Kim HG, Yoon Y, Jeon SY, Yu JA. Change of Visual Perception in Geriatric Strokes after Visuomotor Coordination Training. *Ann. Rehabil. Med.* 2011;35:174–179.
41	Kim 2015	Kim B, Bang D, Shin W. Effects of Pressure Sense Perception Training on Unstable Surface on Somatosensory, Balance and Gait Function in Patients with Stroke. *J. Korean Soc. Phys. Med.* 2015;10:19–27.
42	Kim 2016	Kim M-S. Effect of Robot Assisted Rehabilitation Based on Visual Feedback in Post Stroke Pusher Syndrome. *J. Korea Acad. Coop. Soc.* 2016;17:562–568.
43	Kitisomprayoonkul 2012	Kitisomprayoonkul W. Transcranial Direct Current Stimulation Improves Hand Sensation in Acute Stroke. *Arch. Phys. Med. Rehabil.* [Internet]. 2012;93:e33.
44	Ko 2018	Ko EJ, Chun MH, Kim DY, Kang Y, Lee SJ, Yi JH, Chang MC, Lee SY. Frenkel's exercise on lower limb sensation and balance in subacute ischemic stroke patients with impaired proprioception.

		Neurol. Asia. 2018;23:217–224
45	Koo 2018	Koo WR, Jang BH, Kim CR. Effects of anodal transcranial direct current stimulation on somatosensory recovery after stroke. *Am. J. Phys. Med. Rehabil.* 2018;97:507–513
46	Koohi 2017 (1)	Koohi N, Vickers D, Chandrashekar H, Tsang B, Werring D, Bamiou DE. Auditory rehabilitation after stroke: treatment of auditory processing disorders in stroke patients with personal frequency-modulated (FM) systems. *Disabil. Rehabil.* 2017;39:586–593.
47	Koohi 2017 (2)	Koohi N, Vickers D, Warren J, Werring D, Bamiou DE. Long-Term use benefits of personal frequency-modulated systems for speech in noise perception in patients with stroke with auditory processing deficits: A non-randomised controlled trial study. *BMJ Open*. 2017;7:1–7
48	Krewer 2013	Krewer C, Rieß K, Bergmann J, Müller F, Jahn K, Koenig E. Immediate effectiveness of single-session therapeutic interventions in pusher behaviour. *Gait Posture*. 2013;37:246–250.
49	Lee 2017	Lee J-T, Chon S-C. Does the Addition of Visual Feedback Improve Postural Vertical Training in the Patients with Pusher Syndrome After Stroke? *J. Korean Soc. Phys. Med.* 2017;12:33–42.
50	Lincoln 1985	Lincoln NB, Whiting SE, Cockburn J, Bhavnani G. An evaluation of perceptual retraining. *Disabil. Rehabil.* 1985;7:99–101.
51	McDowell 2019	McDowell N, Dutton GN. Hemianopia and Features of Bálint Syndrome following Occipital Lobe Hemorrhage: Identification and Patient Understanding Have Aided Functional Improvement Years after Onset. *Case Rep. Ophthalmol. Med.* 2019;1–7.
52	Meneghetti 2009	Meneghetti CHZ, Basqueira C, Fioramonte C, Ferracini Júnior LC. Influence of hydrotherapy on trunk control in the pusher syndrome : case report. *Fisioter. e Pesqui.* 2009;16:269–273.
53	Mikołajewska 2012	Mikołajewska E. Posterior pusher syndrome - Case report. *Cent. Eur. J. Med.* 2012;7:354–357.
54	Morioka 2003	Morioka S, Yagi F. Effects of perceptual learning exercises on standing balance using a hardness discrimination task in hemiplegic patients following stroke: A randomized controlled pilot trial. *Clin. Rehabil.* 2003;17:600–607.
55	Nakagawa 1999	Nakagawa N, Akai F, Niiyyama K, Asai T, Tanada M. A case of Peduncular Hallucination after Aneurysmal Subarachnoid Hemorrhage. *No To Shinkei*. 1999;51:65–8.
56	Nakamura 2014	Nakamura J, Kita Y, Yuda T, Ikuno K, Okada Y, Shomoto K. Effects of galvanic vestibular stimulation combined with physical therapy on pusher behavior in stroke patients: a case series. *NeuroRehabilitation*. 2014;35:31–37.
57	Nguyen 2011	Nguyen H, Le C, Nguyen H. Charles Bonnet Syndrome in an elderly patient with acute cerebellar infarction treated successfully with haloperidol. *J. Am. Geriatr. Soc.* 2011;59:761–2.
58	O'Hare 1998	O'Hare AE, Dutton GN, Green D, Coull R. Evolution of a form of pure alexia without agraphia in a child sustaining occipital lobe infarction at 2 1/4 years. *Dev. Med. Child Neurol.* 1998;40:417–420
59	Oppenlander 2015	Oppenlander K, Utz KS, Reinhart S, Keller I, Kerkhoff G. Subliminal

		galvanic-vestibular stimulation recalibrates the distorted visual and tactile subjective vertical in right-sided stroke. *Neuropsychologia*. 2015;74:178–183
60	Papathanasiou 1998	Papathanasiou I, Macfarlane S, Heron C. A case of verbal auditory agnosia: Missing the word...missing the sound... *Int. J. Lang. Commun. Disord.* 1998;33:214–217.
61	Pardo 2019	Pardo V, Galen S. Treatment interventions for pusher syndrome: A case series. *NeuroRehabilitation*. 2019;44:131–140.
62	Park 2015	Park JH, Park JH. The effects of a Korean computer-based cognitive rehabilitation program on cognitive function and visual perception ability of patients with acute stroke. *J. Phys. Ther. Sci.* 2015;27:2577–2579.
63	Poetter 2008	Poetter CE, Vyas BB, Stewart JT, Haley JA. An unusual case of post-stroke Hallucinations *J. Am. Geriatr. Soc.* 2008;56:181–183
64	Rafique 2016	Rafique SA, Richards JR, Steeves JKE. rTMS reduces cortical imbalance associated with visual hallucinations after occipital stroke. *Neurology*. 2016;87:1493–1500.
65	Roberts-Woodbury 2016	Roberts-Woodbury. Visual hallucinations after a stroke. *J. Am. Geriatr. Soc.* 2016;64:s159.
66	Scheets 2007	Scheets PL, Sahrmann SA, Norton BJ. Use of movement system diagnoses in the management of patients with neuromuscular conditions: A multiple-patient case report. *Phys. Ther.* 2007;87:654–669.
67	Tanemura 1999	Tanemura R. Awareness in apraxia and agnosia. *Top. Stroke Rehabil.* 1999;6:33–42.
68	Towle 1990	Towle D, Edmans JA, Lincoln NB. An evaluation of a group treatment programme for stroke patoents with perceptual deficits. *Int. ournal Rehabil. Res.* 1990;13:328–335
69	Voos 2011	Voos MC, Oliveira T de P, Piemonte MEP. Guidelines for assessment and physical therapy treatment in Pusher´s Syndrome: case report. *Fisioter. e Pesqui.* [Internet]. 2011;18:323–328
70	Wang 2016	Wang D, Lin J, Liu X. Effects of visual feedback and core stability training program on post-stroke Pusher syndrome: a pilot randomized controlled study. *Chinese J. Rehab Med.* 2016;31:426–429.
71	Weinberg 1982	Weinberg J, Piasetsky E, Diller L, Gordon W. Treating Perceptual Organization Deficits in Nonneglecting RBD Stroke Patients*. *J. Clin. Neuropsychol.* 1982;4:59–75.
72	Woolf 2014	Woolf C, Panton A, Rosen S, Best W, Marshall J. Therapy for auditory processing impairment in aphasia: An evaluation of two approaches. *Aphasiology*. 2014;28:1481–1505.
73	Yang 2015	Yang YR, Chen YH, Chang HC, Chan RC, Wei SH, Wang RY. Effects of interactive visual feedback training on post-stroke pusher syndrome: A pilot randomized controlled study. *Clin. Rehabil.* 2015;29:987–993.
74	Yun 2018	Yun N, Joo MC, Kim SC, Kim MS. Robot-assisted gait training effectively improved lateropulsion in subacute stroke patients: A single-blinded randomized controlled trial. *Eur. J. Phys. Rehabil. Med.* 2018;54:827–836.
75	Zaharia-Pushkash	Zaharia-Pushkash O, Oleg P, Rodica V, Andrei U. Posterior cerebral

	2010	artery stroke with Balint's syndrome and severe cognitive impairment: clinical and neuroimaging correlation. *Int. J. Stroke*. 2010;5:365–366
76	Zgaljardic 2013	Zgaljardic D, Yancy S, Burton V, Masel B. Auditory Agnosia and Post-Acute Brain Injury Rehabilitation (PABIR): A Case Report. *J. Head Trauma Rehabil.* 2013;28:E35-6
77	Zihl 2000 (1)	Zihl J. Visual agnosia. In: Rehabilitation of visual disorders after brain injury. Hove, UK: Psychology Press; 2000. p. 136–150.
78	Zihl 2000 (2)	Zihl J. Visual agnosia: Balint's syndrome and its treatment. In: Rehabilitation of visual disorders after brain injury. Hove: 2000. p. 122–131
79	Zihl 2000 (3)	Zihl J. Disorders in space perception. In: Rehabilitation of visual disorders after brain injury. Hove, UK: Psychology Press; 2000. p. 110–122.
80	Zihl 2000 (4)	Zihl J. Colour Vision Deficits. In: Rehabilitation of visual disorders after brain injury. Hove: 2000. p. 102–106.

Table S3 Characteristics of included studies, and participant demographics.

	Study count (%)	Population (%)
**Design**		
Case report	36 (45.0)	38 (4.3)
RCT	22 (27.5)	630 (70.5)
N-of-1	9 (11.3)	53 (5.9)
Cohort studies	9 (11.3)	134 (15)
Controlled trial	2 (2.5)	19 (2.1)
Case series	1 (1.3)	5 (0.6)
Case-controlled studies	1 (1.3)	14 (1.6)
**Year**		
1970-1974	2 (2.5)	2 (0.2)
1980-1984	1 (1.3)	35 (3.9)
1985-1989	1 (1.3)	33 (3.7)
1990-1994	6 (7.5)	25 (2.8)
1995-1999	4 (5.0)	4 (0.4)
2000-2004	10 (12.5)	125 (14.0)
2005-2009	5 (6.3)	29 (3.2)
2010-2014	17 (21.3)	196(21.9)
2015-2020	34 (42.5)	444 (49.7)
**Continent**		
Asia	27 (33.8)	414 (46.4)
Europe	26 (32.5)	311 (34.8)
North America	15 (18.8)	81 (9.1)
Australia	7 (8.8)	82 (9.2)
South America	3 (3.8)	3 (0.3)
Not reported	2 (2.5)	2 (0.2)
**Sample size**		
Mean (SD)	11.2 (14.3)	
Median (IQR)	3.5 (1, 16.5)	
Range	1-80	
Studies with n=1 (%)	35 (43.2)	
**Withdrawals**		
0	28 (35.0)	
1	3 (3.8)	
2	2 (2.5)	
4	1 (1.3)	
7	1 (1.3)	
NA	37 (46.3)	
NR	8 (10.0)	
**Lost to follow-up**		
0	26 (32.5)	
1	3 (3.8)	
2	3 (3.8)	
3	2 (2.5)	
NA	37 (46.3)	

NR	9 (11.3)	
**Age**		
Child (<18)	5 (6.3)	5 (0.6)
Adult (18 - 65)	43 (53.8)	517 (57.9)
Older adult (>65)	25 (31.3)	266 (29.8)
Adults and older adults	6 (7.5)	80 (9.0)
Not reported	1 (1.3)	25 (2.8)
**Age**		
Mean (SD)	58.7 (16.4)	
**Sex**		
<25% women	33 (41.3)	144 (16.1)
25 - 50% women	23 (28.8)	447 (50.1)
51 - 75% women	9 (11.3)	228 (25.5)
>75% women	11 (13.8)	12 (1.3)
Not reported	4 (5.0)	62 (6.9)
**Sex**		
Mean % female (SD)	34.8 (33.8)	
**Stroke type**		
Ischaemic	18 (22.5)	61 (6.8)
Haemorrhagic	13 (16.3)	67 (7.5)
Both	20 (25.0)	388 (43.4)
Not reported	27 (33.8)	374 (41.9)
Unclear	2 (2.5)	3 (0.3)
**Stroke severity reported**		
Yes	13 (16.3)	224 (25.1)
No	67 (83.8)	669 (74.9)
**Hemisphere affected**		
0 - 20% right	19 (23.8)	68 (7.6)
21 - 40% right	2 (2.5)	41 (4.6)
41 - 60 % right	9 (11.3)	261 (29.2)
61 - 80% right	10 (12.5)	201 (22.5)
81 - 100% right	29 (36.3)	187 (20.9)
Not reported	11 (13.8)	135 (15.1)
**Timepoint post stroke**		
Acute (up to one month)	19 (23.8)	139 (15.6)
sub-acute (1 to 6 months)	25 (31.3)	340 (38.1)
Sub-acute to chronic	3 (3.8)	31 (3.5)
Chronic (more than 6 months)	20 (25.0)	318 (35.6)
Not reported	13 (16.3)	65 (7.3)

KEY – NA: not applicable; NR: not reported; SD: standard deviation; IQR: interquartile range

Table S4: Definitions of perceptual disorders

Disorder name	Definition
Auditory processing disorder	A hearing problem that affects how the brain interprets sound rather than how sound is carried through the ear to the brain
Charles Bonnet syndrome	Visual hallucinations, which may be simple patterns, or detailed images of events, people or places
Hearing deficit/disorder	A problem in recognizing and interpreting sensory information from the hearing (auditory) system
Proprioceptive deficit/disorder	A problem in recognizing and interpreting sensory information relating to proprioception, or body/joint location and movement
Pusher syndrome	Is characterized by leaning and active pushing toward the contralesional side, affecting posture and possibly leading to instability and loss of balance
Somatosensory deficit/disorder	A problem in recognizing and interpreting sensory information relating to somatosensation, including pressure, temperature or body position.
Tactile deficit/disorder	A problem in recognizing and interpreting sensory information relating touch and information from the skin.
Visual agnosia	A condition in which a person can see but cannot recognize or interpret visual information e.g. an inability to name or describe the use for an object being viewed
Visual hallucination	The visual perception of an object or event in the absence of an external stimulus.
Visual perceptual deficit/disorder	A problem in recognizing and interpreting sensory information from the eyes and visual system
Visual spatial deficit/disorder	A problem in recognizing and interpreting visual information relating to the position of objects, their relation to other objects, and relation to the body, both when at rest and in motion.

Table S5: Summary of intervention description

	NIBS	Pharma-cological	Rehabilitation						Total n (%)
			Compensation	Mixed	Restitution	Substitution	Unclear	With NIBS	
**Total number of interventions**	**7**	**6**	**4**	**20**	**45**	**5**	**4**	**2**	**93 (100)**
**Rationale reported**									
Yes	--	2	2	9	25	3	1	-	**42 (45.2)**
Partial yes	-	1	2	10	13	1	2	-	**29 (31.2)**
No	7	3	-	1	7	1	1	2	**22 (23.7)**
**Materials**									**30 (32.3)**
HCP led	-	-	3	6	21	-	-	-	**28 (30.1)**
Technology	-	-	-	7	17	4	-	-	**13 (14.0)**
Equipment	-	-	-	6	5	1	1	-	**9 (9.7)**
Brain stimulation	7	-	-	-	-	-	-	2	**6 (6.5)**
Pharmacological	-	6	-	-	-	-	-	-	**6 (6.5)**
Not reported	-	-	-	1	2	-	3	-	**1 (1.1)**
Other	-	-	1	-	-	-	-	-	
**Procedure Reported**									
Yes	4	-	1	9	21	1	-	2	**38 (40.9)**
Partial yes	3	3	2	11	22	3	1	-	**45 (48.4)**
No	-	3	1	-	2	1	3	-	**11 (10.8)**
**Who delivered**									
Not reported	5	-	-	16	20	3	3	2	**49 (52.7)**
Physiotherapist	-	-	-	2	17	-	-	-	**19 (20.4)**
Other	-	-	3	1	1	2	1	-	**8 (8.6)**
Occ. Therapist	-	-	1	1	6	-	-	-	**8 (8.6)**
Unclear	-	5	-	-	-	-	-	-	**5 (5.4)**
Researcher	2	-	-	-	1	-	-	-	**3 (3.2)**
Medic	-	1	-	-	-	-	-	-	**1 (1.1)**

**Mode of delivery**									
One to one	7	4	2	18	40	2	1	2	**76 (81.7)**
Not reported	-	2	-	1	3	1	2	-	**9 (9.7)**
Self-delivery	-	-	2	-	-	2	-	-	**4 (4.3)**
Group	-	-	-	1	1	-	1	-	**3 (3.2)**
Unclear	-	-	-	-	1	-	-	-	**1 (1.1)**
**Location**									
Hospital inpatient	1	2	1	6	21	-	2	2	**35 (37.6)**
Not reported	5	3	-	9	10	2	2	-	**31 (33.3)**
Hospital in/out-patient	1	1	1	4	11	2	-	-	**20 (21.5)**
Other	-	-	1	1	1	-	-	-	**3 (3.2)**
Home	-	-	1	-	1	1	-	-	**3 (3.2)**
Hospital outpatient	-	-	-	-	1	-	-	-	**1 (1.1)**
**Duration**									
Less than one week	4	-	-	3	6	1	-	-	**14 (15.1)**
One month or less	-	1	-	7	19	-	-	1	**28 (30.1)**
One- three months	-	-	1	2	4	2	1	-	**10 (10.8)**
More than three months	-	-	1	-	3	-	-	-	**4 (4.3)**
Not reported	3	5	2	8	9	2	3	-	**32 (34.4)**
Unclear	-	-	-	-	4	-	-	1	**5 (5.4)**
**Single / multiple sessions**									
Single session	3	-	-	-	4	1	-	-	**8 (8.56)**
Multiple sessions	3	2	3	16	38	2	2	1	**67 (72.0)**
Not reported	1	4	1	4	3	2	2	-	**17 (18.3)**
Unclear	-	-	-	-	-	-	-	1	**1 (1.1)**
**Personalised?**									
No	3	4	1	10	25	4	4	-	**51 (54.3)**
Partial yes	3	-	-	6	12	-	-	2	**23 (24.5)**
Yes	1	2	-	3	6	-	-	-	**12 (12.8)**
Unclear	-	-	3	1	2	1	-	-	**7 (7.4)**

KEY

NIBS – Non invasive Brain Stimulation

Table S6: Findings from each study

AUTHOR	VERBATIM TEXT QUOTES OF REPORT FINDINGS
An 2019	Comparison of the pre- and post-intervention assessment results showed that both interventions led to the following significant changes: decreased severity of PB scores and increased PASS, BPR, and K-MBI scores (p<.05). In particular, statistical analysis between the two groups, the BLS score was significantly decreased in the GPVT group (p<.05). And PASS, BPR, and K-MBI scores were significantly improved in the GPVT group than in the CPVT group (p<.01, respectively)
An 2020	For the primary outcome, after training, BLS scores were decreased more for the experimental than control group (D = –5.8 vs. D = –4.2, P = 0.002). For secondary outcomes, scores were improved more for the experimental than control group: PASS (D = 13.8 vs. D = 8.5, P < 0.001), BBS (D = 20.1 vs. D = 11.1, P = 0.001), K-MBI (D = 27.0 vs. D = 20.1, P = 0.005), and FMA-L (D = 10.2 vs. D = 6.3, P = 0.002)
Babyar 2018	Six males/4 females age 66, 9.5 standard deviation with admission BLS scores of 5.4 - 3.7 within 8.6 - 8.1 days post-stroke were enrolled. COP-X medial-lateral speed increased for both the tDCS and the GVS protocols compared to sham condition. Fourier Analysis of COP-X velocity for 0-.3 Hz responses showed a significant increase for tDCS stimulation. The.3-1Hz responses for the tDCS condition were decreased from baseline. Lateral thoracic tilt showed significant improvement for tDCS compared to Sham stimulation at 10 minutes and for GVS versus Sham at 15 minutes
Bergmann 2018	RAGT led to a larger reduction of pusher behavior than nRPT at post-test (SCP: U = 69.00, r = −0.33, p = 0.037; Burke Lateropulsion Scale: U = 47.500, r = −0.50, p = 0.003) and at follow-up (SCP: U = 54.00, r = −0.44, p = 0.008). Pusher behavior had ceased in 6 of 15 participants in the intervention group and 1 of 15 participants in the control group at post-test. At follow-up, 9 of 15 and 5 of 15 participants, respectively, no longer exhibited the behavior
Broetz 2004	At day four post-stroke, none of the eight patients were able to sit unsupported. All eight patients showed severe contraversive pushing on each of the Scale for Contraversive Pushing sub-scales. Within three weeks, pushing behaviour improved significantly. When we compared the overall score of the scale for Contraversive Pushing between days four and 24 post-stroke, we found significant improvement (Wilcoxon's Z = –2.23; p = 0.026). Further, at day 24, six of the patients (75%) had recovered sufficiently that they could sit unsupported (McNemar test; p= 0.031). These patients were able to keep a stable upright body position even when distracted; for example, when they did not concentrate on their body orientation. Moreover, 18 days post-stroke all eight patients were able to stand erect while being supported by a physiotherapist. The average degree of pushing behaviour while standing at day 18 was 0.79 on the 'posture' subscale, 0.72 on the 'extension' sub-scale and 1.0 on the 'resistance' sub-scale
Brunsdon 2007	Clear benefits from treatment were evident. The assessment and treatment methods employed provide practical and useful ideas for management of this condition in other children. The results clearly indicate a specific treatment effect for both

	landmark recognition training and specific route-finding training. In other words, constant exposure to the target routes on a day-to-day level did not impact significantly on route finding even over an extended time period, but specific training resulted in significant improvements. In addition, improvements were not restricted to route finding success but were also evident in efficiency of route finding. Although CA had a documented visual agnosia and had difficulty recognising school buildings and landmarks, improving his visual recognition skills was not sufficient for treatment success in actual route finding and orientation
Burr 1970	Mr W appears brighter and more confident in himself and his abilities. His wife reports that he is continuing to do everything for himself at home and although she still does supervise him while getting in and out of the bath, she was not needed to offer any manual assistance
Carey 1993	Graphic and statistical interrupted time-series analyses indicated that treatment produced improvements in seven of eight tactile time series and all four proprioceptive time series. Baseline improvement in one tactile time series prevented unequivocal evaluation of treatment effect. Improvements were clinically significant, discrimination in the affected hand becoming comparable to the other hand and normal performance. Therapeutic effects were maintained at 3-month to S-month follow-up tests
Carey 2005a	Stimulus-specific training was successful for trained texture and proprioceptive discriminations, but it failed to show spontaneous transfer to related untrained stimuli in the same modality in seven of eight experiments in which this was possible. In contrast, intramodality transfer was obtained with stimulus-generalization training in four of five experiments that investigated stimulus-generalization training of texture discrimination. Findings were confirmed by meta-analysis
Carey 2011	Between-group comparisons revealed a significantly greater improvement in sensory capacity following sensory discrimination training, $t(47) = 2.75$, $P = .004$, 1-tailed; mean between-group change = 11.1 SSD; confidence interval 3.0 to 19.2. Improvements were maintained at 6 weeks and 6 months. Sensory discrimination training can achieve significant improvements in functional sensory discrimination capacity after stroke. The clinically oriented training achieved transfer of training effects to novel stimuli. Our findings provide support for introducing sense discrimination training in rehabilitation of sensory deficits after stroke
Carey 2016	Improved touch discrimination of a magnitude similar to previous clinical studies and approaching normal range was found. Patients with thalamic/capsular somatosensory lesions activated pre intervention in left ipsilesional supramarginal gyrus, and post intervention in ipsilesional insula and supramarginal gyrus. In contrast, those with S1/S2 lesions did not show common activation pre intervention, only deactivation in contralesional superior parietal lobe, including S1, and cingulate cortex post intervention. The S1/S2 group did, however, show significant change over time involving ipsilesional precuneus. This change was greater than for the

	thalamic/capsular group (P = .012; d = −2.43; CI = −0.67 to −3.76)
Chen 2011	After 3 weeks, the symptoms of hallucinations and anxiety were relieved. Although some CBS patients might be self-limited without discomfort, low-dose aripiprazole can be considered as a safe medication for significantly anxious patients with CBS
Chen 2012a	indicated that treatment produced improvements in seven of eight tactile time series and all four proprioceptive time series. Baseline improvement in one tactile time series prevented unequivocal evaluation of treatment effect. Improvements The result demonstrated that the Global Processing Training significantly improved visuospatial memory deficits after a right-brain stroke. On the other hand, rote practice without a step-by-step guidance limited the degree of memory improvement. The treatment effect was observed both immediately after the training procedure and 24 h post-training
Cho 2015	Both groups showed significant differences in their relative beta wave values and attention concentration quotients. Moreover, the NFB group showed a significant difference in MVPT visual discrimination, form constancy, visual memory, visual closure, spatial relation, raw score, and processing time. This study demonstrated that NFB training is more effective for increasing concentration and visual perception changes than traditional rehabilitation
Choi 2018	After completion of training, the WVRT group showed significant improvements of+7 (8.25) in theMVPT-3 score, +3.00 (5.25) in the BBS score, and –1.92 (6.33) s in the TUG test, with all results being significantly better than those of the GBT group (P<0.05)
Cogan 1973	These subjective phenomena would come and go with unpredictable intermittency but would lessen in a dimly lit room and would disappear when he closed his eyes or made an attempt to look at them on his left side. They continued for two months but were said to be relieved by Librium
Colombo 2015	Range of motion during shoulder and wrist flexion improved, but only wrist flexion remained improved at 3-month follow-up. These preliminary results suggest that intensive robot-aided rehabilitation may play an important role in the recovery of sensory function. However, further studies are required to confirm these data
Dutton 2017	After a few weeks her simultanagnostic visual dysfunction had regressed almost entirely. She regained her reading skills and visual detection in her peripheral visual field, and returned to full time schooling
Edmans 1991	The results showed little evidence of effective treatment or individual perceptual deficits. Perceptual stimulation alone may have produced some general improvements
Edmans 2000a	There was no significant difference between the treatment groups on patient characteristics or impairments. The results also showed no significant difference between the treatment groups before and after treatment on perceptual ability total

	scores, individual perceptual subtest scores, or functional ability total scores (Mann–Whitney U 642.5–798.0, $p > 0.05$). Wilcoxon matched pairs signed ranks tests showed a significant improvement in both groups after treatment on perceptual and functional
Enders 2013	Vibrotactile noise of all intensities and locations instantaneously and significantly improved Monofilament scores of the index fingertip and thumb tip ($p < .01$). No significant effect of the noise was seen for the Two-Point Discrimination Test scores. Remote application of subthreshold (imperceptible) vibrotactile noise at the wrist and dorsal hand instantaneously improved stroke survivors' light touch sensation, independent of noise location and intensity. Vibrotactile noise at the wrist and dorsal hand may have enhanced the fingertips' light touch sensation via stochastic resonance and interneuronal connections. While long-term benefits of noise in stroke patients warrants further investigation, this result demonstrates potential that a wearable device applying vibrotactile noise at the wrist could enhance sensation and grip ability without interfering with object manipulation in everyday tasks
Fechtel-peter 1990	The stimulation of conscious auditory analysis proved to be increasingly effective over a 4-week period of therapy. We were able to show that the patient's improvement was not only a simple effect of practicing, but it was stable and carried over to non-trained items
Fifer 1993	The most remarkable finding associated with this case is the presence of a unilateral auditory processing disorder when presenting speech materials to the left ear. Intervention for this patient is described in addition to a discussion of possible explanations for the unique pattern of auditory dysfunction
Flint 2005	The positive symptoms abated over a week. The hallucinations may have resulted from infarction, with disinhibition of higher visual centers, or from simple partial seizures not detected by surface EEG. The prolonged symptoms and finding of hypoperfusion by SPECT during the phenomena argue against an epileptiform etiology
Freitas 2017	There is a considerable improvement in FM scores - sensory function and in FIM. The improvement of hemi negligence and SP was also observed by the applied tests. BSE scores and FM, upper limb and lower limb section had minimal differences in the comparison before and after treatment
Fujimoto 2016	We found that GOT thresholds for the affected index finger during and 10 min after the S1 and S2conditions were significantly lower compared with each sham condition. GOT thresholds were not significantly different between the S1 and S2 conditions at any time point
Fujino 2016	At the baseline phase, both scores were poor. Both scores improved after the intervention and follow-up phases, and all the patients could sit independently
Fujino 2019	In both patients, electromyography of the non-paretic triceps brachii muscle revealed excessive activity. To inhibit the excessive activity, ES was applied to the non-paretic biceps muscle. All scores improved after the intervention and

	follow-up phases
Funk 2013	The authors found (a) rapid improvements in trained but also in nontrained spatial orientation tests in all 13 participants, partially up to a normal level; (b) stability of the obtained improvements at 2-month follow-up; (c) interocular transfer of training effects to the nontrained eye in 2 participants suggesting a central, postchiasmatic locus for this perceptual improvement; and (d) graded transfer of improvements to related spatial tasks, such as horizontal writing, analog clock reading, and visuoconstructive capacities but no transfer to unrelated measures of visual performance. Conclusions. These results suggest the potential for treatment-induced improvements in visuospatial deficits by feedback-based, perceptual orientation training as a component of rehabilitation after stroke
Gillen 2003	The child and his family reported that simply having the deficits recognized had been beneficial. The commonsense strategies suggested were incorporated easily into everyday life and school. He is able to attend mainstream school with the help of a support teacher for less than two hours a week. He has achieved greater independence and his self-esteem has improved. Behavioural problems arising from frustration are less frequent. The patient is able to ride his bicycle safely near his rural home. He appeared, therefore, to have the same condition but had adapted to it much more effectively
Gillespie 2019	A standing frame protocol was implemented into standard care to improve CoP. The patient was assisted into a standing frame daily, and the Burke Lateropulsion Scale and Functional Independence Measure were tracked. Improvements in both outcome measures were greater than normative data.
Gottlieb 1991	The role of blinking in reviving the visual percept may be explained accordingly as causing a re-fixation of the target under visual fixation or as resetting the visual pathways for visual processing. When he intentionally blinked the faded visual percept reappeared
Hayashi 2004	We encountered a patient recovering from a right temporal hemorrhage who suddenly developed olfactory illusions and hallucinations in response to certain foods or situations. We discuss the likely mechanism of olfactory hallucinations in this case. About 3 months after the hemorrhage the olfactory episodes had nearly ceased. the patient recovered his appetite and returned to his job
Jahn 2017	The case shows, for the first time, that specific training of disturbed verticality perception can be performed using the Spacecurl and that improvement of SPV (not SVV, which remained unchanged) parallels with the improvement of established measures and with clinical progress. The training method supports active participation of the patient and can be used before the patient is able to stand without or with only limited support. This is limited evidence from a single subject. Currently, a larger series of stroke patients are receiving the same training protocol in a randomized trial
Jamal 2017	In the post-test, a significant reduction in WBA was established in the RBD ($P = 0.009$) and was maintained at D + 15 ($P = 0.01$) and D + 22 ($P = 0.05$), no effect was observed in the LBD. In addition, no significant modification was found on the spatial

	frame in both groups. A significant improvement was found for the Motricity and time up and go in the RBD
Jang 2018	Four months after onset, left leg motor function (Motricity Index [MI] = 51) did not show significant recovery from that at two months after onset (MI = 41); however, in the same period, Nottingham Sensory Assessment and scale for contraversive pushing significantly improved. At four months, the patient was able to stand independently but required manual contact of one person during independent walking on an even floor. At seven months after onset, he was able to walk independently on an even floor
Jo 2012	First, the difference in visual perception function before and after CoTras treatment was analyzed using a paired t-test. As a result of the analysis, there was a statistically significant difference (0.000, $p <.05$). Second, the average AMPS motor skill score increased from 0.90 to 1.11, but there was no statistically significant difference. The average treatment technique score increased from 0.08 to 0.46 points, but there was no statistically significant difference, and clinically the ability to perform daily activities was significantly improved (logit >0.3)
Jokelainen 2000	In the light of the example case, we describe the pusher syndrome that exists previously dealt with only briefly in the Finnish medical literature. A typical cause is a pyramidal tract infarction or bleeding in the brain capsules in the area of the internal crus posterus. The syndrome is characterized by continuous, strong pushing and tilting of the body towards the paralyzed side. Syndrome it is important to identify; it complicates and slows down rehabilitation, especially in the early stages but does not necessarily affect the outcome of rehabilitation. After more than a month and a half, pushing syndrome was so relieved that the patient received permission move in your own room independently without aids Dangerous situations still arose during the turns, stopping and as he walked through the doors - especially if tired. In the doorways he could collide with his left doorstep or get stuck on the left hand to the doorway. Gradually, as walking became more confident, the patient began move outside your room without an assistant. On the way home, score in Berg's balance test (Berg et al. 1989) was 42/56 and on the FIM performance indicator 102/126 and the ten-meter walking time was 8.5 seconds.
Kang 2009	After training, the mean (SD) Motor-free Visual Perception Test score increased significantly in both experimental group (from 65.8 (19.5) to 77.8 (28.7)) and control group (from 68.3 (11.4) to 74.1 (14.8)) (P50.01). Modified Barthel Index score increased significantly in both groups, with the experimental group recording a higher increase. Mean (SD) interest scale score was greater in the experimental group (2.2 (0.8)) than in the control group (1.3 (0.7) (P50.01)
Kim 2011	The intervention group showed significant improvement in visual attention ($p < 0.05$). There was no significant difference in visual memory, visuomotor coordination nd K-DRS ($p > 0.05$). Both groups showed significant increase in K-MMSE and K-MBI scores ($p < 0.05$), but there was no significant difference between the two groups ($p > 0.05$)

Kim 2015a	Experimental groups (group 1 and group 2) showed significant differences in PE, FRT, TUG, and 10-MWT compared to the control group (p <0.05). Group 2 (PSPT on an unstable surface) was significantly different in PE, FRT, and 10-MWT from group 1 (p <0.05). No significant differences were observed for other measures. Pressure sense perception training on an unstable surface might be a significantly more effective method for improving somatosensory function, balance, and walking ability, than PSPT on a stable surface
Kim 2016	The Lokomat group produced significantly better outcomes in SCP (p =0.046), BBS (p =0.046), FI (p =0.038), and TUG (p =0.038) compared with the control group after 4 weeks of intervention. In addition, there were significant correlations between SCP and BBS (p =0.024), FI (p =0.039), and TUG (p =0.030). Lokomat with VR more effectively aided recovery from PS after stroke, and restoration of PS symptoms was related with improvement of balance and gait function
Kitisomp-rayoonkul 2012	Mean (SD) age of control and tDCS groups were 54.7(8.6) and 58. 0(11.9) years, consecutively. Mean (SD) onset were 5.3(1.8) and 9.7(17.8) days. When compared with the control group, the tDCS group significantly improved sensation of hypesthetic hand immediately after tDCS and 30 minutes after cessation (P 0.05). Immediately after stimulation, 50% and 60-70% of tDCS group improved light touch and pinprick sensation, consecutively. Thirty minutes after tDCS cessation, 40% and 50% of the tDCS group improved light touch and pinprick sensation, consecutively. 40-70% of patients have an improvement in 5 out of 7 SWM-tested sites. Most patients in tDCS group had sensation improvement at least 1-level. Most patients in control group had no sensation improvement. Anodal tDCS improves hand sensation in acute stroke immediately after 20-min stimulation. This effect remains at least 30 minutes after stimulation
Ko 2018	Patients in both groups showed significant improvements on the kinesthetic and tactile sensation subscale of the NSA for the lower limb, the K-BBS, the FAC, and the K-MBI, but not the MI, from baseline to post-intervention at 3 weeks. When compared between the two groups, significant improvements were only seen in the kinesthetic sensation subscale of the NSA for the lower limb and the K-BBS (p<0.05). Frenkel's exercise improves sensory and balance recovery among subacute ischemic stroke patients with impaired proprioception and minimal lower limb motor weakness
Koo 2018	Although there was no clear significant difference between the two groups, when the changes from baseline to post treatment evaluation were compared between the groups, a partially significant improvement was observed in the anodal stimulation group compared with the sham stimulation group. Interestingly, the tactile sensation of the unaffected side also improved. Moreover, the greater improvement in activities of daily living function was observed in the anodal stimulation group too. Conclusion: Anodal transcranial direct current stimulation over the primary somatosensory cortex may be a useful adjuvant therapy for the recovery of somatosensation and activities of daily living function in patients with sensory deficits after stroke

Koohi 2017 (a)	The signal-to-noise-ratio (SNR) for 50% correct speech recognition performance was measured with speech presented from 0° azimuth and competing babble from ±90° azimuth. Spatial release from masking (SRM) was defined as the difference between SNRs measured with co-located speech and babble and SNRs measured with spatially separated speech and babble. The SRM significantly improved when babble was spatially separated from target speech, while the patients had the FM systems in their ears compared to without the FM systems
Koohi 2017 (b)	Speech reception thresholds showed clinically and statistically significant improvements in intervention but not in standard care subjects at10 weeks in aided and unaided conditions
Krewer 2013	Compared to PT-vf, Lokomat therapy had a significant effect on the BLS of pusher patients but no significant effect on the SCP values. GVS had no significant effect on these values on either scale. BLS is more useful than SCP to detect small changes for clinical trials and routine treatment. Forced control of the upright position during locomotion seems to be an effective method for immediately reducing the pushing behaviour of stroke patients, probably because it recalibrates a biased sense of verticality, via the somatic graviception. This finding, however, does not allow prediction of its long-term effects. Furthermore, it would be interesting to evaluate repetitive, multi-session DGO therapy and the amount of therapy needed to effectively reduce the pusher behaviour
Lee 2017	Compared to the average score at baseline, the average SCP score for the SPV training without visual feedback decreased from 5.3 to 2.8, from 4.6 to 3, and from 3.5 to 2.7 for subjects 1, 2, and 3, respectively. However, the average score for the SPV training with visual feedback decreased from 5.3 to 3.1, from 4.6 to 3.5, and from 3.5 to 3.3 for subjects 1, 2, and 3, respectively
Lincoln 1985	No significant differences were found between the groups either before or after 4 weeks of treatment on measures of visual perception or on ADL scales
McDowell 2019	The patient suffered spontaneous left occipital lobe brain hemorrhage from a ruptured arteriovenous malformation. This was surgically excised. Short lived right upper limb intermittent jerking, with additional left sided weakness, ensued. Anomalous EEG recordings, with right-sided bias, arose from the posterior temporo parietal area. A right homonymous hemianopia was evident. During the ensuing 17 years she experienced multiple complex difficulties, until, at a lecture describing how to identify and support children with CVI, she realized she herself had many of the difficulties described. Visual assessment identified hemianopia and dorsal stream dysfunction. Discussion. Following identification, characterization, and explanation of the impact of her visual difficulties, she both gained greater awareness of her visual difficulties and their impact and developed a range of strategies leading to functional improvement of her visual field loss and amelioration of her dorsal stream dysfunction, with great improvement in quality of life. Following identification, characterization, and explanation of the impact of her visual difficulties, she both gained greater awareness of her visual difficulties and their impact and developed a range of strategies leading to functional improvement of her visual field loss and amelioration of her dorsal stream dysfunction, with great improvement in quality of life. Her new knowledge and understanding of her

	condition have considerably improved her quality of life because she can now use rational approaches to make the best use of her vision
Meneghetti 2009	Aquatic physiotherapy consisted of two weekly sessions of one hour for two months, totaling 16 sessions, using the methods Bad Ragaz and Halliwick, to strengthen the musculature of the trunk and the upper limbs, respectively. In the evaluation after the intervention, important reduction in the head inclination angles (from 31.7º to 10.6º), the shoulders (from 10.3º to 1.7º) and the trunk (from 9.6º to 3.0º). The program of aquatic physiotherapy, therefore, provided the participant with the syndrome of sensitive pusher improves trunk symmetry and alignment
Mikola-jewska 2012	The author describes, on the basis of the literature and own research, symptoms and methods of the treatment of the little-known posterior pusher syndrome. After two weeks (ten sessions) of the therapy there was an observed extinction of symptoms of the posterior pusher syndrome (SCP score = 1.25, both sitting and standing, Table 2). Previously reported tendency to fall backwards could no longer be demonstrated
Morioka 2003	Twenty-six subjects completed the study. Data indicate that more parameters indicating postural sway were significantly decreased in the experimental group than in the control group. Also, there was a significant difference between the groups in change scores (pre-exercise minus post exercise) of length and enveloped area. The plantar perception exercise used as a method in this study is considered to be effective as a supplemental exercise for standing balance. The possibility of clinical application using the hardness discrimination task with rubber as a balance exercise is therefore suggested
Nakagawa 1999	Hyperdynamic therapy relieved the visual hallucinations
Nakamura 2014	In both patients, the SCP scores were reduced only during phase B2. Although the BLS scores improved at the A1 phase, a larger improvement was seen at the two B phases. Multisession GVS combined with physical therapy may have positive effects on PB in clinical setting
Nguyen 2011	The patient had previously presented to his ophthalmologist with visual acuity complaints and was diagnosed with hypertensive retinopathy and bilateral cataracts but neglected to mention these ongoing visual hallucinations for fear of being diagnosed with mental illness. As a diagnosis of exclusion, CBS was confirmed, and the patient was reassured but also treated temporarily with haloperidol nightly until immediate marked resolution of hallucinations
O'Hare 1998	The progress of cognitive visual dysfunction over an 8-year period of a child who sustained bilateral occipital-lobe infarctions at the age of 2% years is described. She survived with normal intelligence and went on to attend mainstream school. She manifested many features of cognitive visual impairment and, in particular, developed a form of pure alexia without agraphia. She achieved some letter-by-letter reading but no sight vocabulary development, including to her own name. She learned to write imaginatively employing phonetically true spelling but cannot read what she has written. Her progress and the dif5culties encountered during the management of her condition are discussed in this first case report of the evolution of pure alexia without agraphia in childhood. The features of this syndrome in the

	developing child who has never developed the capacity to read are contrasted with that seen in affected adults
Oppen-laender 2015	Both groups performed these tasks under three experimental conditions on three different days: a) sham GVS where electric current was applied only for 30 s and then turned off, b) left-cathodal GVS and c) right-cathodal GVS, for a period of20 min per session. Left-cathodal GVS, but not right-cathodal GVS significantly reduced all parameters in the SVV. Concerning STV GVS also reduced constant error and range numerically, though not significantly. These effects occurred selectively in the impaired patient group. In conclusion, we found that GVS rapidly influences post stroke verticality deficits in the visual and tactile modality, thus highlighting the importance of the vestibular system in the multimodal elaboration of the subjective vertical. Left-cathodal GVS, but not right-cathodal GVS significantly reduced all parameters in the SVV. Concerning STV GVS also reduced constant error and range numerically, though not significantly. These effects occurred selectively in the impaired patient group. In conclusion, we found that GVS rapidly influences post stroke verticality deficits in the visual and tactile modality, thus highlighting the importance of the vestibular system in the multimodal elaboration of the subjective vertical
Papathan-asiou 1998	The neuropsychological nature of verbal auditory agnosia is not fully understood. This study aims to describe the language deficits and the remediation strategies used in a person with verbal auditory agnosia. In addition, it will address the theoretical issues concerning the nature of the phenomenon and the clinical implications in the management of people with this disorder
Pardo 2019	All five participants demonstrated improvements in pushing behavior, balance and transfer status. These outcomes provide preliminary evidence of decreased pushing behavior, and improved balance and transfers following a program of interventions designed to improve the functional outcomes of patients with PS. Larger studies are needed to confirm these findings, and whether these interventions are effective for patients with less severe pushing behavior
Park 2015	Therapy Cognitive Assessment (LOTCA) and Motor-free Visual Perception Test-3 (MVPT-3) were performed. [Results] Both groups showed significant improvement in LOTCA and MVPT-3. Furthermore, there were significant differences in LOTCA and MVPT-3 between the two groups. [Conclusion] CBCR with CoTras may contribute toward the recovery of cognitive function and visual perception in patients with acute stroke
Poetter 2012	There is no literature regarding the treatment of asomatognosia, but the use of established cognitive rehabilitation techniques for neglect seemed reasonable and ultimately led to the complete resolution of the asomatognosia and the hallucinations and marked improvements in neglect
Rafique 2016	Increased application of rTMS corresponded with a reduction in intensity of visual phosphene hallucinations and was reflected in altered blood oxygen level-dependent (BOLD) signal. fMRI revealed focal excitatory discharges at the border of

	the lesion, highlighting the origin of phosphenes. Post-rTMS, rTMS did not simply suppress activity in the patient but rather re-distributed the previously imbalanced cortical activity not only at the stimulation site but in remote cortical regions so that it more closely resembled that of controls
Roberts-Woodbury 2016	Charles Bonnet syndrome is a syndrome of release, or visual, hallucinations due to stroke or secondary to visual impairments, including cataracts, macular degeneration, and glaucoma. Patients with this syndrome usually realize that the hallucinations are not real. Many patients are afraid to mention the hallucinations to their doctors for fear of this symptom being mistaken for a psychiatric illness. Charles Bonnet syndrome should be considered in the differential diagnosis for visual hallucinations. It is, unfortunately, a diagnosis of exclusion. The visual hallucinations worsened during his hospitalization, and he was placed on a trial of Risperidone. This medication was stopped due to side effects, and over the next few days, the hallucinations began to improve without any other pharmacological intervention
Scheets 2007	Use of movement system diagnoses may have multiple benefits for patient care. The possible benefits include decreasing the variability in management of patients with neuromuscular conditions, minimizing the trial-and-error approach to treatment se-lection, improving communication among health care professionals, and advancing research by enabling creation of homogenous patient groupings
Tanemura 1999	Measurable recovery of visual perception was achieved through activities planned to reorganize his visual perception with intact kinesthetic information
Towle 1990	Treatment had no discernable effect on measures of cancellation or cube copy. In 3/6 patient there was some improvement in Rey complex figure scores, but in only one was this distinct and attributable to treatment
Voos 2011	After treatment, the patient was reassessed and showed improvement in all scales. The assessment of the pushing symptom and the the manual Jebsen-Taylor function tests were those that registered the highest percentages improvement, 79% and 46%, respectively. The protocol used, even though it was started six months after the injury, provided perceptual and functional improvement, which suggests the importance of physical therapy in the recovery of SP
Wang 2016a	A comparison of pre and post treatment assessment revealed that the three experimental groups led to increase in the balance scores and Barthel Index, but the core stability training group hadn't obvious change in Pusher syndrome scores. Visual feedback training group, visual feedback and core stability training group had obvious decrease in Pushers syndrome scores ($p<0.01$) with no obvious difference between these two groups ($p>0.05$)
Weinburg 1981	The remaining 18 patients were re-examined after 1 month and served as controls. The design of the treatment program was based on the hypothesis that non-neglecting RBD patients fail to appreciate and synthesize elements of complex visual material due to a breakdown in compensation for a persistent lateral bias in visual-spatial attention. Upon post testing, it was found that those patients who received training exhibited significantly improved performance, as compared

	to controls, on a subgroup of visuo-cognitive tasks. These results are discussed in terms of: (1) offering indirect support for the argument that pathological asymmetries in attention play a significant role in the failure of RBD patients on many visuo-cognitive tasks; and (2) offering a basis for extending the treatment of perceptual problems in RBD patients. Upon post testing, it was found that those patients who received training exhibited significantly improved performance, as compared to controls, on a subgroup of visuo-cognitive tasks. These results are discussed in terms of: (1) offering indirect support for the argument that pathological asymmetries in attention play a significant role in the failure of RBD patients on many visuo-cognitive tasks; and (2) offering a basis for extending the treatment of perceptual problems in RBD patients
Woolf 2014	Group analyses showed no significant changes in tests of word and non-word discrimination as a result of therapy. One comprehension task improved following therapy, but two did not. There was also no indication that therapy improved the discrimination of treated words, as assessed by a priming task. The facilitation scores indicated that participants needed less support during tasks as therapy progressed, possibly as a result of improved listening. There was a significant effect of time on the telephone message task. Across all tasks there were few individual gains
Yang 2015	A comparison of pre- and post-training assessment results revealed that both training programs led to the following significant changes: decreased severity of pusher syndrome scores (decreases of 4.0 ±1.1 and 1.4 ±1.0 in the experimental and control groups, respectively); improved balance scores (Increases of 14.7 ±4.3 and 7.2 ±1.6 in the experimental and control groups, respectively); and higher scores for lower extremity motor control (increases of 8.4 ±2.2 and 5.6 ±3.3 in the experimental and control groups, respectively). Furthermore, the computer-generated interactive visual feedback training program produced significantly better outcomes in the improvement of pusher syndrome ($p < 0.01$) and balance ($p < 0.05$) compared with the mirror visual feedback training program. Although both training programs were beneficial, the computer-generated interactive visual feedback training program more effectively aided recovery from pusher syndrome compared with mirror visual feedback training
Yun 2018	After intervention, the experimental group showed greater improvement in the BLS score at T1 (experimental group: Δ=-1.9, control group: Δ=-1.1, P=0.032) and T2 (experimental group: Δ=-2.8, control group: Δ=-6.5, P<0.001) than the control group. In addition, the BBS was significantly improved in the experimental group at T1 (experimental group: Δ=+7.1, control group: Δ=+1.9, P<0.001) and T2 (experimental group: Δ=+13.0, control group: Δ=+6.1, P<0.001). There were significant between-group differences in the PASS at T1 (experimental group: Δ=+3.2, control group: Δ=+1.6, P=0.014) and T2 (experimental group: Δ=+8.8, control group: Δ=+4.3, P<0.001). RAGT ameliorated lateropulsion and balance function more effectively than CPT in subacute stroke patients. Early RAGT may be recommended for patients with lateropulsion after stroke

Zaharia-Pushkash 2010	We can conclude that patient showed good clinical improvement in regard to motor deficit and partial compensation of visuospatial disorders, but no improvement in mnestic functions. Follow up examinations revealed progressive deterioration of cognitive status
Zgaljardic 2013	The current case report demonstrates functional improvement of a patient with acquired auditory agnosia following her participation within a residential PABIR program despite a lack of discernable change in focal neurological impairment
Zihl 2000 (1)	After treatment, both patients showed a considerably better discrimination performance...The improvement in colour discrimination also became evident in the recognition of coloured object and in colour naming. Both patients benefitted from the improvement in colour vision. Patient 1 reported being able once again to compare and select, at least in part, colour hues on textiles
Zihl 2000 (2)	The patient could not only fixate a target and shift fixation more accurately after practice; her scanning behavior also improved…She was able to reach accurate for the door handle, to pick food up accurately with the fork, and to put on makeup using a mirror. When the accuracy of visual reaching for objects was compared before and after training, the patient performed much better. She reported a distinct improvement in reaching for objects in her everyday life. After practice, the patient required less time to read correctly even longer words, and could now also read short sentences
Zihl 2000 (3)	Despite considerable difference between patient, saccadic accuracy and fixation were found to be markedly improved when compared with the recording before practice. …saccadic accuracy was found to be increased. The field of oculomotor scanning was found to be considerably enlarged after training, but all patients still exhibited signs of visual disorientation, at least in the dot pattern condition. After treatment, patients also showed an improved visual recognition performance for objects and scenes. Most importantly, all three patients were now able to find their way in familiar surroundings, although all required relatively long time to find rooms, objects etc.
Zihl 2000 (4)	Both patients gained an effective oculomotor compensation. In addition to the improvement in trained object class, there was some generalisation to other classes of visual objects, except for letters and familiar faces. We found considerable improvement in the identification of all characters. There was evidence of further improvements at 6 months

Key

PB, pusher behaviour; PASS, Postural Assessment Scale for Stroke; BPR, Balance Posture Ratio; K-MBI, Korean-modified Barthel Index; BLS, Burke Lateropulsion Scale; GPVT, game-based postural vertical training; CPVT, conventional postural vertical training; BBS, Berg Balance Scale; FMA-L, Fugl-Meyer Motor Assessment-Lower Extremity; COP-X, seated haptic centre of pressure; GVS, galvanic vestibular stimulation; tDCS, transcranial direct current stimulation; RAGT, robot-assisted gait training; nRPT, nonrobotic physiotherapy; SCP, Scale of Contraversive Pushing; SSD, Standardized Somatosensory Deficit; CBS, Charles Bonnet Syndrome; NFB, neurofeedback training; MVPT, Motor Free Visual Perception Test; TUG, Timed Up and Go Test; GBT, general balance training; EEG,

electroencephalogram; SPECT, Single-Photon Emission Computerized Tomography; FM, Fugl-Meyer; FIM, Functional Independence Measure; SP, pusher syndrome; GOT, grating orientation task; ES, electrical stimulation; CoP, contraversive pushing; SPV, subjective postural vertical; SVV, subjective visual vertical; RBD, right brain damage; LBD, left brain damage; VR, virtual reality; CON, control group; WMFT, Wolf Motor Function Test; SD, standard deviation; AMPS, Assessment of Motor and Process Skills; K-DRS, Korea Dementia Rating Scale; K-MMSE, Korean Mini Mental Status Exam; K-MBI, Korean modified Barthel Index; PE, Pressure Error; FRT, The Balancia Function Reach Test; 10-MWT, 10m Walking Test; PSPT, pressure sense perception training; K-BBS, Korean Berg Balance Scale; FAC, Function Ambulation Classification; MI, Motricity Index; NSA, Nottingham Sensory Assessment ; PT-vf, physiotherapy with visual feedback components; ADL, Activities of Daily Living; CVI, Cortical Visual Impairment; rTMS, Repetitive Transcranial Magnetic Stimulation; CPT, conventional physiotherapy; PABIR, post-acute brain injury rehabilitation
